# Research Advances of Injectable Functional Hydrogel Materials in the Treatment of Myocardial Infarction

**DOI:** 10.3390/gels8070423

**Published:** 2022-07-06

**Authors:** Wei Hu, Cui Yang, Xiaodan Guo, Yihong Wu, Xian Jun Loh, Zibiao Li, Yun-Long Wu, Caisheng Wu

**Affiliations:** 1 Fujian Provincial Key Laboratory of Innovative Drug Target Research and State Key Laboratory of Cellular Stress Biology, School of Pharmaceutical Sciences, Xiamen University, Xiamen 361102, China; 32320211154316@stu.xmu.edu.cn (W.H.); 32320201154126@stu.xmu.edu.cn (X.G.); wuyihong@xmu.edu.cn (Y.W.); 2 School of Medicine, Xiamen University, Xiamen 361003, China; yangcui@stu.xmu.edu.cn; 3 Institute of Materials Research and Engineering (IMRE), Agency for Science, Technology and Research (A*STAR), Singapore 138634, Singapore; lohxj@imre.a-star.edu.sg; 4 Institute of Sustainability for Chemicals, Energy and Environment (ISCE^2^) Agency for Science, Technology and Research (A*STAR), Singapore 138634, Singapore; 5 Department of Materials Science and Engineering, National University of Singapore, 9 Engineering Drive 1, Singapore 117576, Singapore

**Keywords:** myocardial infarction, hydrogel, stem cells, growth factor, genes, drug

## Abstract

Myocardial infarction (MI) has become one of the serious diseases threatening human life and health. However, traditional treatment methods for MI have some limitations, such as irreversible myocardial necrosis and cardiac dysfunction. Fortunately, recent endeavors have shown that hydrogel materials can effectively prevent negative remodeling of the heart and improve the heart function and long-term prognosis of patients with MI due to their good biocompatibility, mechanical properties, and electrical conductivity. Therefore, this review aims to summarize the research progress of injectable hydrogel in the treatment of MI in recent years and to introduce the rational design of injectable hydrogels in myocardial repair. Finally, the potential challenges and perspectives of injectable hydrogel in this field will be discussed, in order to provide theoretical guidance for the development of new and effective treatment strategies for MI.

## 1. Introduction

Cardiovascular diseases are the main cause of human death, while myocardial infarction (MI) is the main cause of the high incidence of ischemic heart disease [1,2,3]. MI is defined as sudden ischemic death of myocardial tissue, which is myocardial necrosis caused by acute and persistent ischemia and hypoxia of coronary arteries. Although percutaneous coronary intervention improves early survival, the patient population at risk of heart failure expands [4,5]. Epidemiological studies have shown that the increased incidence of heart failure after infarction has paralleled the decline in acute mortality in recent decades, which will significantly increase the high socio-economic burden and affect the quality of life of patients who survive acute myocardial infarction [6]. There are three main treatments for MI: drug therapy, left ventricular assist device implantation, and heart transplantation. Although the mortality of MI has decreased, the risk of donor heart transplantation and trauma is limited [7]. Furthermore, the ventricular remodeling is negative, which leads to the progressive decline of the cardiac function of myocardial infarction survivors, and there are still some limitations such as irreversible myocardial necrosis and cardiac function. Therefore, it is an important subject of cardiovascular disease research to develop new effective strategies for treating MI.

Nowadays, cardiac tissue reconstruction, prevention of ventricular remodeling and scar tissue formation, and reduction of heart failure and heart rupture have become the focus of new effective strategies [8,9], including the development and application of a variety of biomaterials. Currently, biomaterials developed to promote ischemic myocardial repair include patches, hydrogels, scaffolds, and particles [10,11,12]. It is worth mentioning that hydrogel has the following advantages. (1) It has good biocompatibility and low immunogenicity, and its three-dimensional cross-linked spatial structure might replace damaged myocardium to bear ventricular stress. Injectable hydrogels can be modified to further improve their biological properties, such as mechanical properties and electrical conductivity, enabling them to mimic native myocardium. (2) It can deliver all kinds of active cells, growth factors, drugs, and genes to specific local areas of heart tissue. (3) It can be administered through minimally invasive surgery, which can reduce surgical trauma. (4) It shows the advantages of regeneration and degradation [13,14,15,16,17,18].

Based on the above information, this review aims to focus on the role of hydrogel materials especially injectable hydrogels in the treatment of MI and to explore the therapeutic mechanism of injectable hydrogel combined with stem cells, cytokines, drugs, and genes. Last but not least, the existing problems of injectable hydrogel and the perspectives of using hydrogel formulations in the treatment of MI will also be discussed.

## 2. Biological Mechanism of Myocardial Infarction

**Figure 1:**
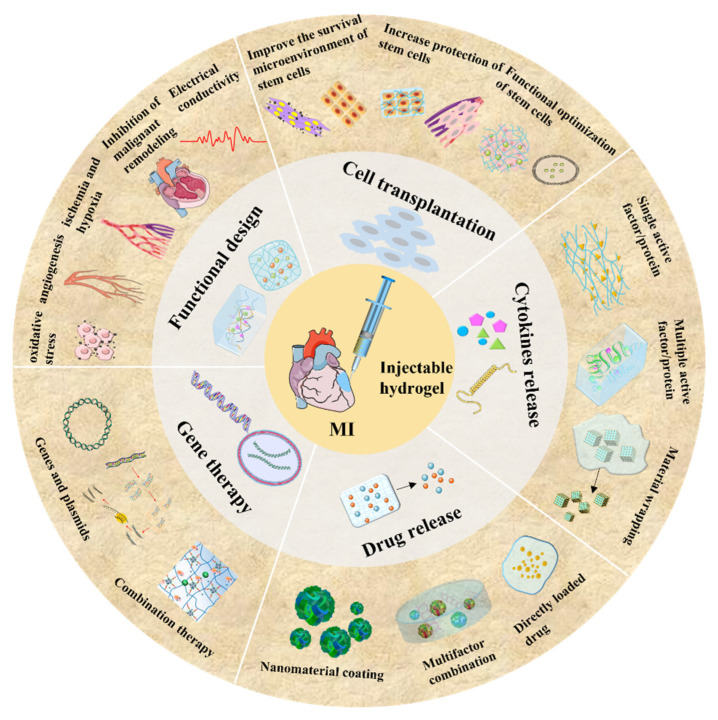
Schematic summary of injectable functional hydrogel materials for the treatment of myocardial infarction.

Coronary atherosclerosis will result in a narrowing of the lumen and insufficient blood supply. When collateral circulation is not fully established and blood supply is sharply reduced, myocardial ischemia lasting more than 1 h will lead to MI [19]. After MI, with the decrease of ATP (adenosine triphosphate) level in the endocardium, ischemic heart or susceptible myocardial cells can no longer maintain their structural integrity [20]. In this case, ultrastructural changes occur, resulting in irreversible damage, which eventually leads to apoptosis or necrosis of myocardial cells. In the healing stage, MI has gone through the stages of inflammation, proliferation, and healing [21]. As shown in Figure 1, in the inflammatory phase, the generation of ROS (reactive oxygen species) activates the complement system, stimulates the expression of P-selectin, and activates the NF-κB (nuclear transcription factor-κB) system to upregulate the synthesis of cytokines and chemokines [22]. Releases pro-inflammatory mediators IL-1 (interleukin-1), (TNF)-α (tumor necrosis factor-α), and IL-6 (interleukin-6), which further upregulates the expression of CXC and CC chemokines, thereby promoting leukocyte chemotaxis, triggering an inflammatory cascade, while further helping to clear necrotic cells and cellular matrix debris [23,24]. Subsequently, negative regulation of TLR (toll-like receptor) signaling, etc., induces and releases anti-inflammatory molecules thereby transitioning to the proliferative phase. Such as TGF-β (transforming growth factor-β), IL-10 (interleukin-10), aldosterone’s broad lipid-derived mediators, and VEGF (vascular endothelial growth factor), etc., thereby activating anti-inflammatory pathways [25]. Cardiac fibroblasts are transformed into myofibroblasts, and the ECM (extracellular matrix) gradually deposits proteins, during which angiogenesis in the infarcted area provides oxygen and nutrients to the metabolically active mesenchymal cells in the healing infarcted area [26,27]. As the infarcted area heals, a new myocardial matrix is gradually established, scarring is formed, the ventricle dilates to maintain cardiac output and cardiac repair transitions to maturity. Healing of the infarct is intertwined with the geometric remodeling of the chamber, with dramatic changes in ventricular geometry, dilation of the chamber, and thinning of the infarcted portion, leading to ventricular remodeling [28].

**Figure 2:**
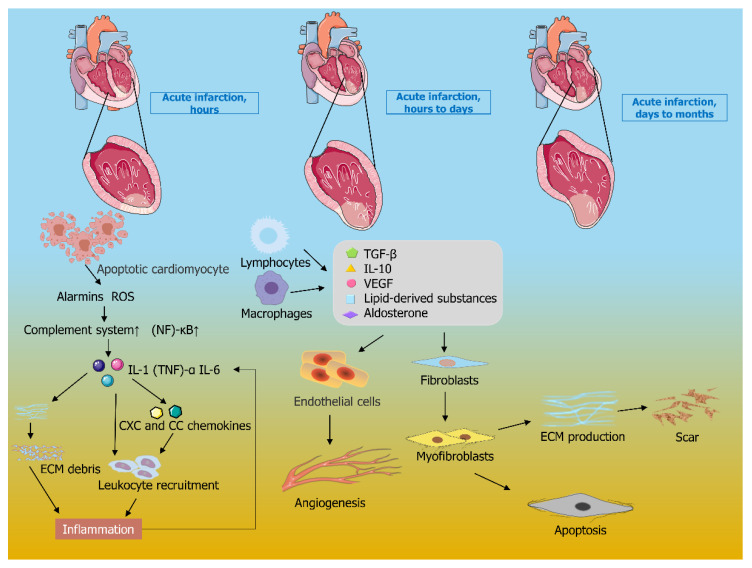
Biological progression after myocardial infarction.

The pathological process after MI is a dynamic development process as shown in Figure 2, and the related cardiac tissue cells and factors have biological roles in different stages. Therefore, according to the pathological mechanism of different stages, it requires a rational design of hydrogel materials in the treatment process, in order to conform to the pathological process of MI. In the inflammatory stage, it is a feasible way to improve the curative effect of the hydrogel by inhibiting myocardial apoptosis, promoting degradation or removal of infarct matrix fragments, and reducing the over-expression of pro-inflammatory factors. In the proliferation stage, a hydrogel strategy should be designed to promote the maturation and angiogenesis of fibroblasts, which might be beneficial to the healing of infarction. In the mature stage, functional hydrogels could be designed to prevent excessive ventricular dilatation and to reduce fibrosis or scar formation, in order to effectively prevent complications, such as heart failure.

## 3. Design of Functional Injectable Hydrogels

Hydrogels are useful for drug delivery and tissue engineering [29,30,31,32,33,34,35,36,37]. An injectable hydrogel, made of hydrophilic polymers, can be injected in situ into a specific area of myocardial tissue in the form of a solution, with the ability to increase the thickness of the ventricular wall and reshape the left ventricular geometry [38,39]. A sol-gel phase transition occurs upon stimulation, which provides mechanical support for myocardial tissue and promotes myocardial tissue repair [40,41]. As a typical example, a previous report has shown that the synthesized high modulus hydrogel can improve the mechanical properties of the hydrogel by changing the substitution amount of methacrylic acid in the macromolecular polymer of hyaluronic acid methacrylate [42]. More importantly, it could reduce the pressure on the ventricular wall due to poor ventricular remodeling and show smaller infarct expansion and left ventricular dilation. Furthermore, pig myocardial stroma cells after processing could be effectively maintained within in vivo and in vitro myocardial hydrogel matrix at the temperature of 37 °C [43]. This bioactive hydrogel design could achieve in situ injection in the myocardium of a rat, while it could simulate the natural extracellular environment of the myocardium. In this case, endothelial cells and smooth muscle cells could in vivo and in vitro migrate to the myocardial matrix [44]. As a result, this bionic ECM hydrogel, which significantly improves cardiac function, has good safety and therapeutic effectiveness [45]. For the other case, injection of synthetic recombinant human collagen type I (RHCI) matrix at the late proliferative stage after MI has successfully restored cardiac mechanical properties and reduced scar size [46], which maintained distal wall thickness and prevented heart enlargement. This hydrogel design provides a bionic platform for safe and effective endogenous tissue repair. Experimental evidence has shown that cardiac function was restored by promoting healing, cardiomyocyte survival, and reducing pathological remodeling of myocardium [47]. At present, hydrogel with alginate as the main component has been applied in the clinical treatment of MI. More interestingly, its therapeutic potential of inhibiting the injury process after MI has been proved in phase I and Phase II clinical trials, while the safety of implantation has also been effectively verified [48,49,50]. The above studies confirmed the preliminary feasibility and the potentials of injectable hydrogel as implantable scaffolds for cardiac tissue engineering [51].

**Figure 3:**
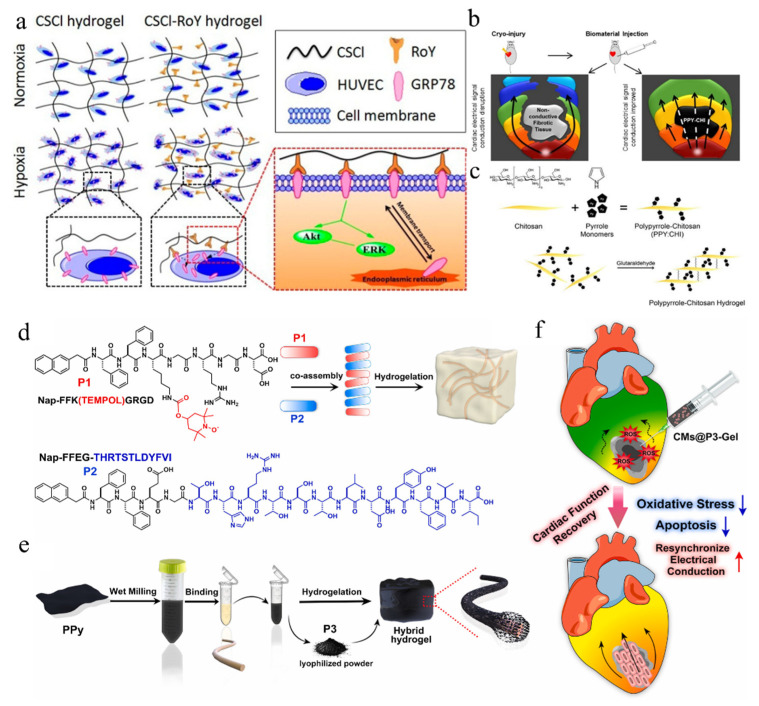
(**a**) Effect of CSCl-RoY hydrogel on the Akt and ERK signal transduction pathways. (Reprinted with permission from Ref. [57]. 2015, American Chemical Society). (**b**) Generation of a polypyrrole-chitosan hydrogel [58]. (**c**) Intra-myocardial injection of PPy: CHI following cardiac injury improved electrical impulse propagation of scar tissue in vivo [58]. (**d**) The chemical structures of peptides and their hydrogelation by co-assembly. (Reprinted with permission from Ref. [59]. 2022, Elsevier). (**e**) The process to prepare the ROS-scavenging/conductive composite hydrogel. (Reprinted with permission from Ref. [59]. 2022, Elsevier). (**f**) The illustration of the ROS-scavenging/conductive composite hydrogel to repair myocardial infarction. (Reprinted with permission from Ref. [59]. 2022, Elsevier).

Although injectable hydrogel can provide physical stability to infarcted myocardium, structural enhancement and improved biocompatibility might not be good enough to maintain cardiac functions over a long time [50]. Therefore, nowadays, more experimental design ideas are focused on expanding the structure and properties of hydrogel materials [13,52]. A number of in-depth studies were conducted on the mechanism of repairing MI with injectable hydrogel, so as to achieve better repair effects on infarcted myocardium [53]. As a typical example, intra-myocardial injection of chitosan hydrogel was experimentally illustrated to control infarction narrow scope, to retain wall thickness, and to improve heart function. In detail, this chitosan hydrogel could be beneficial for the removal of ROS and the recruitment of chemokines, with the ability to improve the MI microenvironment, to promote the implantation, survival, or homing of stem cells in the ischemic heart, and to accelerate myocardial repair [54]. However, the regulation of chitosan hydrogel on the MI microenvironment is mainly achieved by scavenging ROS, and its effect is relatively limited. Meanwhile, the mechanism of how chitosan hydrogel improves the MI microenvironment, promotes stem cell implantation and survival, and promotes neovascularization and cardiac repair is still unclear [54,55]. Therefore, it is necessary to further explore the possibility in repair function by chitosan hydrogels on myocardium. For this purpose, a thermosensitive CSCl-RoY (chitosan chloride-RoY) hydrogel, connected with RoY peptide to CSCl chain through amide bonds, was designed. RoY is a 12 amino acid synthetic peptide (YPHIDSLGHWRR) that specifically binds to the GRP78 (78-kD glucose-regulated protein) receptor, which is mainly expressed on the membrane surface of vascular endothelial cells during hypoxia [56]. Studies have shown that the RoY peptide-modified hydrogels can promote angiogenesis in the hypoxia state after MI and improve cardiac function [54,57]. Compared with CSCl hydrogel only, CSCl-RoY hydrogel can promote the survival, proliferation, migration, and tubular formation of human umbilical vein endothelial cells under hypoxia. In this study, the mechanism was investigated. Under hypoxia conditions, RoY ligand in CSCl-RoY hydrogel interacts with the GRP78 (glucose-regulated protein78) receptor on the surface of human umbilical vein endothelial cells, inducing the endogenization of GRP78 receptor. In this case, the expression upregulation of p-AKT (phosphorylated protein kinase B) and p-ERK1/2 (phosphorylated extracellular signal-regulated kinase 1/2) was detected, and the signaling pathway of cell survival/proliferation was activated (Figure 3a). In addition, starting from the myocardial conductance pathway, the scar after MI blocks the transmission of electrical pulse and delays local contraction, leading to ventricular dysfunction. Furthermore, in previous studies, conductive PPy (polypyrrole) was coupled to chitosan skeleton to prepare a conductive biomaterials [58]. After intramuscular injection, electrical pulse propagation of scar tissue in the body was promoted and synchronous ventricular contraction was reconstructed. Its principles are shown in Figure 3b,c based on the similar design ideas mentioned above. In short, when injected with hydrogel alone, we divided the function of hydrogel in the treatment of myocardial infarction into the following categories: (1) to reduce oxidative stress; (2) to promote angiogenesis; (3) to alleviate ischemia and hypoxia in the infarct area; (4) to inhibit the malignant remodeling caused by metalloproteinases; and (5) to increase the electrical conductivity of myocardium.

### 3.1. To Reduce Oxidative Stress

ROS is a highly reactive oxygen derivative. As a byproduct of aerobic cell metabolism, ROS acts as a second messenger of signal transduction in regulating calcium processing mechanisms and contractile proteins [60,61]. After MI, excessive ROS acts on protein kinases including calmodulin-dependent protein kinase II, resulting in myocardial hypertrophy [60]. Secondly, when the level of ROS is high, oxidative stress might lead to apoptosis or necrosis of myocardial cells. In this case, the reduced nicotinamide adenine dinucleotide phosphate oxidase containing NOX2 (nicotinamide-adenine dinucleotide phosphate (NADPH) oxidases 2) is involved in the process of myocardial remodeling and contraction dysfunction after MI [62]. Based on the idea of reducing the damage caused by oxidative stress, in a large animal model of MI, researchers have demonstrated that injection of a temperature-responsive NIPAAm (N-isopropylacrylamide)-mPEGMA (polyethylene glycol methyl methacrylate) copolymer hydrogel with ROS scavenging properties can reduce ROS, increase wall thickness, and improve MI peripheral function [63]. Hydrogels with the function of scavenging ROS can effectively protect the myocardium from oxidative damage in vivo, such as significantly reducing cell membrane peroxidation and apoptosis, inhibiting inflammation, preserving cardiac function, and promoting left ventricular angiogenesis [64]. ROS increases dramatically after ischemia-reperfusion after MI [65], resulting in channel activity and Ca^2+^ increased conductance [66]. In this case, intracellular Ca^2+^ increased stimulation of Ca^2+^ dependent on biochemical pathways, which leads to the development of myocardial hypertrophy. Additionally, intracellular Ca^2+^ elevated levels also lead to an increase in mitochondrial Ca^2+^ uptake and mitochondrial ROS levels [61,67], which is positive feedback and plays a key role in the progression of cardiac hypertrophy [66,68]. Furthermore, Ratcliffe et al. used nanoparticles mediated dual transmission of antioxidant curcumin and anti-L-type Ca^2+^ channel peptide, which reduced mitochondrial metabolic activity by scavenging ROS and disrupting cytoskeletal communication between LTCC (L-type Ca^2+^ channels) and mitochondria [69]. Curcumin and peptide could synergistically reduce ischemic reperfusion damage through different mechanisms, and this combination therapy will be more effective than treatment alone.

### 3.2. To Promote Angiogenesis in Infarct Area

One of the methods to treat ischemic tissue after MI is to enhance microvascular perfusion by delivering pro-angiogenic signals. VEGF is one of the most effective pro-angiogenic factors [70]. For example, Stuppd et al. developed a small peptide hydrogel that mimics VEGF for the treatment of MI in vivo [71]. The hydrogel displayed a peptide that mimics VEGF on the surface of nanofibers, which showed enhanced signal and biological activity by activating specific vascular endothelial growth factor receptors. It is found that VEGF could promote angiogenesis better than endothelial cells. In another case, citrate polymer hydrogel synthesized by citric acid and polyethylene glycol diol was injected into the left ventricle of myocardial infarction rats [72]. The citrate degradation by-products promoted angiogenesis and cardiac protection, by activating the PI3K-Akt-mTOR (phosphatidylinositol 3′-kinase-Akt-mammalian target of rapamycin) pathway and manipulating the citric acid cycle. With the effective release of MYDGF (myeloid-derived growth factor), this self-degradable hydrogel with an angiogenic effect might have great potential as medical biomaterials.

### 3.3. To Alleviate Ischemia and Hypoxia in Infarct Area

In addition to oxidative stress, hypoxia is one of the inflammatory characteristics of the tissue microenvironment after severe ischemic myocardial infarction [73,74]. Extremely low oxygen content in the infarct area is the main cause of massive myocardial cell death. At present, it is necessary to develop a method that can continuously deliver oxygen to the MI area [75], and a number of studies have prepared a method that can remove ROS and generate O_2_ with injectable hydrogel for the treatment of MI [76]. For example, a multi-functional hydrogel under the condition of hypoxia and inflammation can not only remove ROS, but also continue to produce oxygen, with the ability to suppress apoptosis, to increase the proportion of M1/M2 macrophages, to promote angiogenesis, to reduce the effect of the infarction area, thus to regulate MI or adverse tissue microenvironment, and to improve heart function.

### 3.4. To Inhibit Malignant Remodeling Caused by Metalloproteinases

After MI, MMPs (matrix metalloproteinases) are activated, and the imbalance between MMPs and TIMPs (tissue inhibitor of matrix metalloproteinases) leads to left ventricular remodeling after MI [77,78]. Under normal physiological conditions, endogenous MMPs inhibition is achieved by the synthesis and release of TIMPs [79], and the relative TIMP level might not increase in the early period after MI, resulting in an imbalance between endogenous proteolytic activity and inhibition [77]. Therefore, a number of studies used hyaluronic acid hydrogel to locally deliver recombinant rTIMP-3 (recombinant tissue inhibitor of metalloproteinases-3) to promote the proliferation of myofibroblasts in the infarction area and effectively block the adverse remodeling after MI [80]. Containing MMPs shearable peptide-crosslinked HA(hyaluronic acid) based hydrogels that are responsive to MMPs [81], rTIMP-3 achieved controllable release and significantly attenuated key indicators of ventricular remodeling, such as left ventricular dilation and thinning of the infarct area at 28 d after MI.

### 3.5. To Increase Electrical Conductivity of Myocardium

Injectable hydrogel provides structural support for the injured hearts, while conductive injectable hydrogel enhances biological conduction, synchronizes heart contractions, and improves therapeutic effects [82,83]. Zhou et al. introduced GO (graphene oxide) nanoparticles into OPF (oligo (poly (ethylene glycol) fumarate)) hydrogels to prepare a conductive hydrogel, which not only provides mechanical support for the infarct area but also electrically connects isolated cardiomyocytes to intact tissue. In this case, the tissue with synchronize contraction could restore ventricular function and maintain cardiac function, in comparison with injections of non-conductive polymers [84]. At the same time, this study showed that OPF/GO hydrogel can upregulate Cx43 (connexin 43) and gap junction-related protein production, by providing mechanical support and electrical connection between healthy myocardium and cardiomyocytes in scar and by activating Wnt signaling pathways. Furthermore, homogeneous double network hydrogels [85], composed of a rigid/hydrophobic/conductive network of chemically crosslinked PTAA (polythiophen-3-acetic acid) and a flexible/hydrophilic/biocompatibility network of crosslinked MAAG (methacrylate aminated gelatin), could be prepared to tunable properties. In detail, this double network hydrogel can adjust the swelling and mechanical and electrical properties by tuning the ratio of the PTAA network and MAAG network. More importantly, this double network hydrogel can promote the survival and proliferation of BADSCs (brown adipose-derived stem cells) and improve the efficiency of cardiac differentiation of BADSCs. The expression of Cx43 was up-regulated and electrical stimulation could further ameliorate the effect. This hydrogel can be used as an ideal scaffold for cardiac tissue engineering with good mechanical properties. More importantly, hydrogels with excellent electrical conductivity and anti-fatigue properties have strong benefits for cardiac repair [86,87]. The hydrogel obtained by combining the above different functions could also greatly increase the therapeutic efficiency of myocardial infarction [51,88]. The oxidant TEMPOL was integrated into the peptide, and an injectable ROS scavenging/conducting composite hydrogel was constructed through the specific binding between the conducting PPy and the multicomponent co-assembled peptide. As shown in Figure 3d–f, this therapeutic strategy, which combines increasing myocardial conductance and scavenging ROS, can significantly promote cardiac repair [59].

## 4. Hydrogel as a Carrier for Transplanted Cells

At present, a number of studies have shown that stem cell transplantation technology can be used to repair myocardial injury and improve cardiac function, including ECSs (endogenous cardiac stem cells) [89], HSCs (hematopoietic stem cells) [90], ESCs (embryonic stem cells) [91], MSCs (mesenchymal stem cells) [92], and IPSCs(induced pluripotent stem cells) [93,94], etc. However, the treatment effect is poor due to the lack of adhesion of the transplanted cells. The mechanical loss caused by the beating of the heart, and the poor microenvironment in the infarct area, lead to the low retention rate and survival rate of the transplanted cells [89,95,96]. As an injectable polymeric formulation, hydrogel can provide a three-dimensional growth environment for cells, improve the survival rate of transplanted cells, and significantly improve cardiac function [97]. For example, Christman et al. demonstrated that fibrin hydrogel increased the survival rate of skeletal muscle myoblast transplantation [98], reduced infarct size, and increased blood flow to the ischemic myocardium. In a study of self-assembling peptide NFs (nanofibers) loaded with BMNCs (bone marrow mononuclear cells) [99], NFs injections improved cell retention and improved cardiac function at 28 d after myocardial infarction. Furthermore, these two formulations played a synergistic role in improving cardiac function. BMNCs injections significantly increased systolic function after myocardial infarction but not diastolic function, while combined injection of BMNCs and NFs improved contractility and relaxation function. It is worth mentioning that the performances of hydrogel transplanted stem cells in the treatment of myocardial infarction relied on its formulations. For example, by using hyaluronic acid hydrogel encapsulating BMNCs in the treatment of rat myocardial infarction [100], hyaluronic acid hydrogels have been experimentally proved to prevent mechanical loss and loss of nest apoptosis cells. However, cell apoptosis caused by myocardial ischemia hypoxia was not disturbed. Currently, it is still a challenge to promote the long-term survival of transplanted stem cells in hydrogel. Furthermore, it became more difficult for stem cell survival, upon the appearance of coronary myocardial infarction area of ischemia hypoxia, insufficient blood supply and subsequent inflammation, and myocardial substrate degradation, after the occurrence of myocardial infarction. In this case, it might also be valuable to explore the optimal design of cell encapsulated hydrogel designs, by considering the strategy to improve the transplanted cells survival environment, to increase the protective effect of stem cells, and to achieve the optimization of stem cell function.

### 4.1. To Improve the Survival Microenvironment of Stem Cells

#### 4.1.1. To Scavenge Reactive Oxygen Species

**Figure 4:**
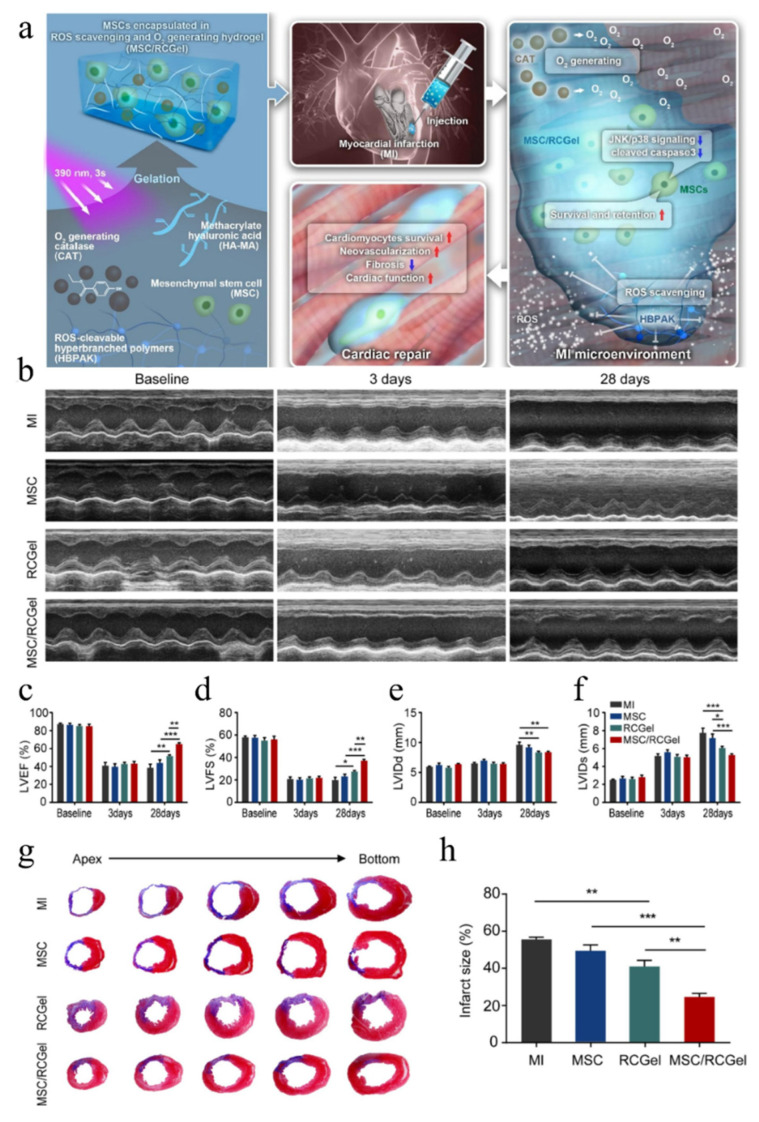
(**a**) Scheme of mesenchymal stem cells-loaded ROS-scavenging and O_2_-generating hydrogel (MSC/RCGel) for MI treatment. (**b**) Representative echocardiography images, and quantitative analysis of (**c**) left ventricular ejection fraction (LVEF), (**d**) left ventricular fractional shortening (LVFS), (**e**) left ventricular end diastolic diameter (LVIDd) and (**f**) left ventricular end systolic diameter (LVIDs) before MI (baseline), three days and 28 days after MI. (**g**) Representative Masson’s trichrome staining images in five different sections from the apex to the bottom of the heart 28 days after MI. (**h**) Quantitative analysis of the infarcted size. (Reprinted with permission from Ref. [106]. 2022, Elsevier). Level of significance (*—*p* < 0.05, **—*p* < 0.01, ***—*p* < 0.001).

The pathological process of myocardial infarction, especially the initial inflammation of myocardial infarction, is closely related to ROS [101]. In detail, cardiomyocytes and fibroblasts could be induced to produce a series of ROS, in case of local myocardial ischemia and hypoxia after coronary artery infarction, reperfusion injury caused by vascular recalculation, or intra-muscular injection of hydrogels. These ROS are mainly O_2_^−^, H_2_O_2_, HO_2_·, ·OH, which could cause serious damage to myocardial cells and exogenous transplanted stem cells [102,103]. To prevent this undesired effect, a thermosensitive CSCl-GSH (chitosan chloride glutathione) hydrogel was designed to effectively scavenge superoxide anion [104], hydroxyl free radical, or DPPH free radical, and to inhibit oxidative stress injury or apoptosis of cardiomyocytes. In 2017, Wang et al. prepared a series of fullerenol/alginate saline gels with antioxidant activity [105], which can prevent the adverse signal transduction in cells after myocardial infarction and reduce the oxidative stress damage caused by ROS after myocardial infarction. Furthermore, the experimental results showed that fullerenol/sodium alginate hydrogel can inhibit JNK (c-Jun N-terminal kinase) signaling pathway, activate ERK (extracellular regulated protein kinases) and p38 (P38 MAPK) signaling pathways, reduce cell apoptosis, and improve the survival or proliferation of BADSCs. As shown in Figure 4a, MSCs were encapsulated in a hydrogel consisting of ROS-scavenging HBPAK (hyperbranched polymers), O_2_-generating CAT (catalase), and HA-MA (methacrylate hyaluronic acid). MSCs can maintain good viability, inhibit the activation of ROS-induced JNK/p38 apoptosis signaling pathway, and greatly improve the survival rate under oxidative stress conditions [106]. More importantly, the apoptosis level of mesenchymal stem cells also decreased in a hypoxia environment. As a result, mesenchymal stem cells were able to survive in this anti-ROS and anti-hypoxia composite, thereby enhancing the viability of the infarcted heart and promoting angiogenesis (as shown in Figure 4b–h). At present, some hydrogel materials with good electrical conductivity have a good effect in combination therapy with loaded stem cells [107]. While promoting myocardial conductance, they can also play a role in scavenging ROS to promote stem cell survival. A modified graphene oxide nanomaterial, which is both an electroactive component and can greatly scavenge ROS [108]. When this material was incorporated into silk protein hydrogels, binding to stem cells improved cardiac repair efficiency.

#### 4.1.2. To Simulate Extracellular Matrix

Injection of a bionic matrix into the myocardial infarction area not only significantly improves cardiac function, but also has certain safety and can regulate the physiological activity of stem cells [51,109]. For example, the synthesis of IGF-1C (insulin-like growth factor C) structure domain polypeptide chitosan hydrogel was conducted and was proved to simulate the microenvironment for stem cell survival [110]. This hydrogel provided good cell adhesion, migration, and proliferation of the microenvironment, thereby enhancing the activity of stem cells. Yao et al. demonstrated that such hydrogel could promote the proliferation, anti-apoptosis, and angiogenesis of human PMSCs (placenta-derived mesenchymal stem cells) in vitro [111]. Combined intramyocardial injection with PMSCs could improve the survival of PMSCs in the myocardial microenvironment. Meanwhile, a combined injection could promote angiogenesis in the infarcted area and reduce inflammation, prevent undesired infarct tissue fibrosis, and improve cardiac function. In addition, Yoon et al. used self-assembled biodegradable PA (peptide amphiphilic) nanomaterials to improve the therapeutic effect of PSCs (Pluripotent Stem Cells) derived CMs (cardiomyocytes) [112] and introduced a fibrin-derived cell adhesion ligand (RGDS) into PA to promote cell adhesion and survival. The incorporation of NMP-2 (nuclear matrix protein 2) biodegradable sequences enables the scaffold to be gradually degraded and replaced by the natural ECM produced by surrounding cells. This design mimicked the physical and biochemical complexity of natural ECM. In another case, folic acid peptide hydrogels were proved to mimic the natural ECM, with no immunogenicity and cell toxicity [113]. Compared with the traditional peptide hydrogel, this composite hydrogel had better gelling properties and biocompatibility and could provide a comfortable environment in vivo for IPSCs. It significantly improved the retention and survival of IPSCs cells in vivo, increased the number of IPSCs cells with the ability to differentiate into cardiomyocytes, and amplified the effect of IPSCs on angiogenesis through differentiation and paracrine.

### 4.2. To Promote Stem Cell Protection

#### 4.2.1. To Improve Retention of Hydrogel-Loaded Cells in the Infarcted Area

It has been studied that when loaded with cells, hydrogels are less satisfactory in modulating the therapeutic effect of delivered stem cells and have a lower ability to support the adhesion and proliferation of encapsulated cells [114]. To solve this issue, Wang et al. modified pNIPAAM (Poly (N-isopropylacrylamide)) hydrogels with SWCNTs (single-walled carbon nanotubes) [115]. PNIPAAm/SWCNTs hydrogels showed high bioactivity on BASCs (bone marrow mesenchymal stem cells), by promoting BASCs cell adhesion and proliferation, in comparison with PNIPAAm hydrogels alone. An injectable natural polymer hydrogel with electrical conductivity as a cell delivery vehicle not only has excellent biocompatibility and cell delivery ability for UCMSCs (umbilical cord mesenchymal stem cells), but more importantly, can promote the growth of UCMSCs, proliferation, and differentiation, thereby improving damaged myocardial tissue and rebuilding myocardial function [116]. The addition of chemokines to the hydrogel, such as SDF-1α (Stromal cell-derived factor-1 alpha), which promotes homing of stem cells in the infarcted area, also could increase their retention [117]. In addition, injection of ECM hydrogels encapsulating MSCs (mesenchymal stem cells) by means of IPC (intrapericardial cavity) injection can significantly solve the problem of low cell retention. The results showed that MSCs had a higher cardiac retention rate than IM injection when delivered by the IPC route, and the heart repair capacity of the infarcted rats was enhanced [118].

#### 4.2.2. To Alleviate Ischemia and Hypoxia for Nutrient Provision

After MI, the infarct area is severely ischemic and hypoxic, and the cell survival rate is poor under hypoxic conditions. In this case, establishing a continuous oxygen delivery system in the infarct area might greatly improve the survival rate of transplanted stem cells and cardiomyocytes [75,119]. An oxygen release system has been prepared for in situ oxygen release in myocardial infarction [120], which consisted of hydrogen peroxide (H_2_O_2_) released microspheres, catalase, and an injectable thermosensitive hydrogel. The design was utilized to improve the retention of microspheres and stem cells in cardiac tissue during the myocardial injection. Microspheres are based on polylactic PLGA (poly (lactide-co-glycolide)), PVP (poly (2-vinlypyrridione)) and H_2_O_2_. The breakage of microspheres by catalase might produce oxygen, which lasted for at least two weeks, in a similar manner to myocardial infarction (1% O_2_). In this case, the introduction of oxygen release in hydrogel significantly enhanced cell survival, with no cell death observation after 7 d culture. Cells even grew after 7 d and realized cardiac differentiation. In addition to the release of oxygen by hydrogen peroxide, in situ oxygen production was also studied by using an oxygen-producing hydrogel composed of CaO_2_ (peroxide calcium) loaded with GelMA (gelatin methacryloyl), which could produce oxygen under hypoxic conditions (1% O_2_) over 5 d and was sufficient to relieve the metabolic stress of the cardiac side population cells coated in this composite hydrogel [121]. More importantly, this composite hydrogel design significantly improved cell survival, compared to GelMA-only formulation.

Improving oxygen partial pressure is another way to increase oxygen content in the infarct area. Guan et al. developed a family of PFC (perfluorocarbon)-coupled hydrogels that could increase oxygen partial pressure to improve cell survival under hypoxic conditions, and these hydrogels have high oxygen preservation and fast gelation properties [122]. Fast gelation enables rapid solidification of the hydrogel, which could effectively fix cells in tissues and increase cell retention rate. The experimental results showed that under hypoxic conditions (1% O_2)_, the partial pressure of oxygen of the hydrogel without PFC (pO_2_ = 28.1 ± 2.9 mm Hg) was significantly lower than that of DPBS (Dulbecco’s Phosphate-Buffered Saline) (pO_2_ = 35.0 ± 3.1 mm Hg), while the partial pressure of oxygen was significantly greater than that of DPBS after adding 10% PFC to the hydrogel (pO_2_ = 75.8 ± 8.2). MSCs were encapsulated in PFC hydrogels and cultured under 1% O_2_ for 14 days. The cells survived and proliferated, while the cells in the control group hydrogels died massively. Hypoxic myocardial infarction areas can also be widely used.

### 4.3. Functional Optimization of Stem Cells

#### 4.3.1. To Enhance the Biological Activity of Stem Cells

The addition of active factors into the cell growth environment could enhance the biological function of the original stem cells. For example, by using biotin-streptomycin affinity, IGF-1 (insulin-like growth factor 1) could be linked to the self-assembly peptide. CPCs (cardiac progenitor cells) have an IGF-1-IGF-1 receptor system, which can promote their survival and growth [123]. Combined intracardial injection of CPCs and this self-organized polypeptide nanofiber hydrogel showed that the volume of regenerated cardiomyocytes increased and the infarct size decreased [124,125]. HE-cad-Fc (Human E-cadherin fusion protein) can effectively promote the paracrine activity of MSCs (esenchymal stem cells). PLGA (poly (lactic-coglycolic acid)) microparticles modified with hE-cad-Fc were combined with hMSCs (human mesenchymal stem cells) to construct functionalized MSC aggregates. Encapsulating them into injectable hydrogels could effectively improve the MI microenvironment compared to the direct encapsulation of MSCs by hydrogels [126]. On the other hand, adenovirus could be utilized to transfect MSCs by genetic modification. HGF (hepatocyte growth factor) could also be beneficial to MSCs [127], with significant apoptosis reduction and MSCs growth promotion. Furthermore, cytokines could also promote the differentiation of stem cells. Chan et al. transplanted FGF-10 (fibroblast growth factor-10) with self-assembled polypeptide nanofibers in combination with ESCs or IPSCs into the infarct area of mice [128]. The experimental results showed that ESCs and IPSCs cells could differentiate into cardiac cells. For the other case, a nitric oxide release controlled hydrogel, which is catalyzed by β galactosidase, was found to promote the secretion of angiogenic cytokines by AD-MSCS (adipose-derived β-mesenchymal stem cells) [129]. Thus, by promoting angiogenesis and AD-MSCS survival, this hydrogel improves cardiac function after MI by enhancing AD-MSCS implantation and paracrine effect. However, the field is still in its infancy and needs to study the role of more cytokines in stem cell differentiation.

#### 4.3.2. Application of Exosomes

The paracrine effect of stem cells plays an important role in promoting the survival and angiogenesis of cardiomyocytes in the infarcted area, while the paracrine effect is mainly mediated by EV (extracellular vesicles) [130], which could be exosomes as the most abundant sub-vesicles. Compared with stem cells, stem cell-derived exosomes not only have the same beneficial functions but also have the ability to prevent the trigger of immune responses and to reduce the heterologous risk of implantation. In this case, exosomes might serve as another effective strategy for the treatment of ischemic cardiovascular disease [131,132]. For example, exosomes with high miR-675 (micro ribonucleic acid-675) content were obtained by transfecting exosome-derived mother cells with miR-675 molecules, in which miR-675 inhibited senescence and damage-induced phenotypes by targeting the TGF-β1-Smad2/3 (transforming growth factor β1-small mothers against decapentaplegic 2/3) signaling pathway [133]. The exosomes were encapsulated with a functional polypeptide hydrogel, while its myocardial injection inhibited inflammatory response and fibrosis levels, significantly improving cardiac function [134]. Since the immune system plays a central role in both the inflammatory and repair phases after MI [135], it has been found that DCs (dendritic cells) derived DEXs (exosomes) are involved in antigen presentation, immune activation, and inhibition. It is worth mentioning that DEXs can improve cardiac function through CD4^+^ T cells [136]. DEXs were combined with alginate hydrogel [137]. After myocardial injection, the hydrogel had better effects on immune regulation, anti-apoptosis, promotion of angiogenesis, and increase in infarct wall thickness [138]. This report also explored the mechanism of DEXs in improving cardiac function. DEXs have been found to activate regulatory Tregs (Regulatory T cells), which further regulates macrophage polarization and enables macrophages to metastasize to M2-type cells. This immunotherapy will be a novel strategy for treating myocardial infarction [138]. In addition, the exosomes were combined with a conductive hydrogel that matches the conductivity of the native myocardium to obtain the conductive Gel@Exo system. It can significantly improve the interaction between cells, promote cell proliferation and angiogenesis, and have a significant therapeutic effect on MI [139].

## 5. Hydrogels as Factor/Protein, Drug, Gene Release Carriers

Upon the combination of hydrogel with functional factor/protein, drug, and gene for myocardial infarction treatment, it mainly plays a role in local delivery and controllable release. Injectable hydrogel forms a stable solid scaffold in the infarct area after coagulation, and the wrapped factor/protein, drug, and gene are continuously released to avoid their rapid metabolism [140,141,142]. Injectable hydrogel systems can out-performance traditional drug delivery methods, due to their advantages such as low invasiveness, high efficiency, and sustained local delivery [41,50].

### 5.1. For Loading with Bioactive Factors and Proteins

#### 5.1.1. Loading with Single Active Factor/Protein

The treatment of myocardial infarction by stem cells is mainly due to the paracrine role of stem cells secreting various bioactive factors in the myocardium [143], such as VEGF, PDGF (platelet-derived growth factor), SDF-1 (stromal cell-derived factor-1) [144,145], etc. The introduction of bioactive factors is one of the approaches to treat myocardial infarction. The continuous release of hydrogel had a slow-release effect on factors and proteins in myocardial infarction. For example, FGFs (fibroblast growth factors), a family of growth factors initially identified to promote fibroblast growth, promote cardiac repair through pro-angiogenesis, anti-apoptotic, and survival mechanisms [146]. As a bioactive factor deposit, ROS-sensitive cross-linked polyvinyl alcohol hydrogels were prepared, with the ability to respond to the microenvironment where excessive ROS were generated after myocardial ischemia-reperfusion injury or myocardial infarction [147]. This hydrogel was experimentally proved to show bFGF (basic fibroblast growth factor) delivery ability and experienced fast degradation in a ROS-rich environment for myocardial repair. This design achieved enhanced retention of BFGF in the pericardium and controllable bFGF release into the heart muscle in an “on-demand” manner.

A number of proteins and peptides can also play a certain role in promoting cardiac function recovery [148,149]. For example, angiopoietin-1 (Ang1) binds to integrin and activates the presurvival pathway to promote cardiomyocyte survival, while short sequence QHREDGS peptide is the binding site of Ang1 and integrin to support cardiomyocyte adhesion and survival [150]. Therefore, researchers tried to fix the pro-survival peptide QHREDGS on the chitosan collagen hydrogel as an integrin-binding ligand, and interacted with the integrin of cardiomyocytes [151]. Experimental results showed that this design significantly improved the shape and function of the heart, including a 53% improvement in scar thickness, a 34% reduction in the scar area, a 35% improvement in shortened fraction, and a 62% improvement in ejection fraction.

#### 5.1.2. Multiple Active Factor/Protein Combination Therapy

Microangiogenesis is one of the effective methods to inhibit ventricular remodeling and improve cardiac function after myocardial infarction. However, vascular regeneration is a complex process requiring the participation of many factors, and a single factor might lead to vascular leakage and immaturity [152]. VEGF can promote the proliferation and migration of endothelial cells, so as to promote the generation of new blood vessels in the infarction area, and to inhibit myocardial fibrosis in late myocardial infarction [153]. In the late stage of angiogenesis, PDGF-BB (platelet-derived growth factor-BB) could stimulate the smooth muscle cells to the newly formed blood vessels, and promote the perfection of the maturity, and function of blood vessels [154]. It has been proved that alginate hydrogel combined with VEGF and PDGF-BB could induce angiogenesis and function improvement, in comparison with single factor treatment only after myocardial infarction [155]. By designing VEGF embedding fibrin gel and PDGF embedding heparin-based coagulant, the system provides a rapid release of VEGF, while the slow and sustained release of PDGF upon single injection [156]. In comparison with the single factor approach, this composite drug delivery system is effective in improving cardiac function, ventricular wall thickness, angiogenesis, myocardial survival, and reducing fibrosis or inflammation in the infarct area. Therefore, the matching of active factors with different properties and the controlled release of different components in different stages will make them play a synergistic role in myocardial infarction and greatly increase the therapeutic efficiency [157].

**Figure 5:**
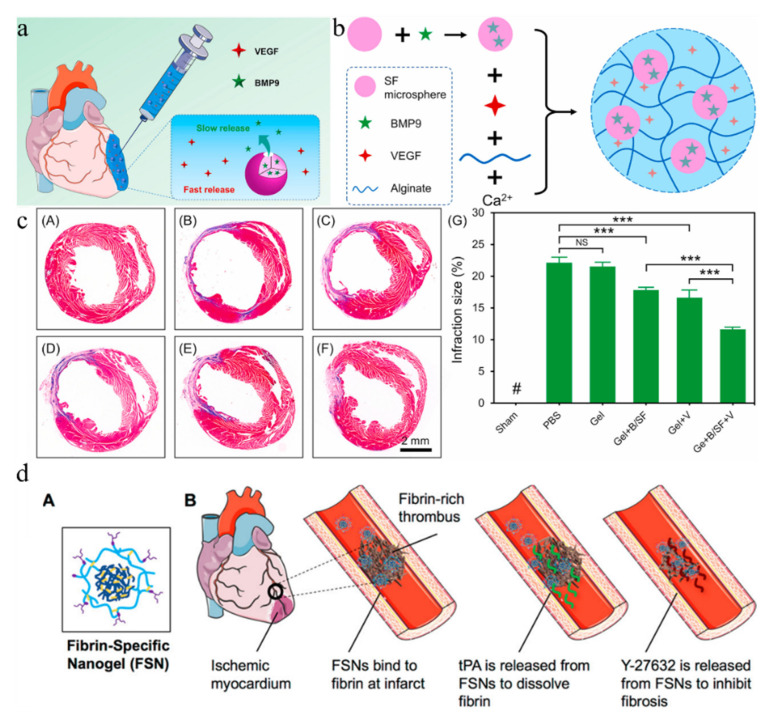
(**a**) Hydrogel-loaded BMP9 and VEGF in the treatment of myocardial infarction [159]. (**b**) Schematic of the preparation process of the composite hydrogel [159]. (**c**) Assessment of fibrosis in the infarct area 28 days post-MI and quantitative analysis of the infarction size as expressed by the ratio of infarct area to total left ventricular area. (**A**–**F**) Representative images of infarcted sections performed by Masson’s trichrome staining. (**A**) Sham, (**B**) PBS, (**C**) Gel, (**D**) Gel + B/SF, (**E**) Gel + V, (**F**) Gel + B/SF + V. (**G**) Quantitative analysis of the infarction size as expressed by the ratio of infarct area to total left ventricular area. [159]. (**d**) (**A**) Fibrin-specific nanogel design. (**B**) drug-loading FSNs bind to fibrin at the site of infarction, releasing fibrinolytic drugs and small molecule cell contractile inhibitors to alleviate cardiac fibrosis (Reprinted with permission from Ref. [160]. 2018, American Chemical Society). Level of significance (***—*p* < 0.001, NS—not significant).

In addition to combination therapy between cytokines, they can also work together with bioactive proteins. For example, BMP9 (bone morphogenetic protein 9) can effectively slow down the process of myocardial fibrosis by inhibiting the action of TGF-β1 (transforming growth factor β1) [158]. By taking these advantages, SF (silk fibroin) protein microspheres were used as the carrier of BMP9. These microspheres were added into the alginate containing VEGF and crosslinked with calcium gluconate to form a composite hydrogel [159]. This composite hydrogel can rapidly release VEGF to promote angiogenesis in the early MI and continuously release BMP9 to inhibit fibrosis formation in the long MI. In this case, the biological activity of proteins could be maintained to fit the timings of each stage of myocardial infarction, which effectively accelerated angiogenesis in MI mouse models, inhibited fibrosis formation, and enhanced cardiac function (Figure 5a–c).

#### 5.1.3. Material Wrapping Factor/Protein with Improved Release Performance

Nanomaterials and microspheres with bioactive factors wrapping or adsorption could be mixed into hydrogel to prolong the diffusion time and treatment effectiveness, to improve the myocardial repair rate, and to produce more favorable effects on tissue repair. For example, SF protein microspheres were prepared by a microfluidic device. IGF-1 was physically adsorbed onto SF microspheres. These microspheres were loaded into alginate brine gel to enhance the sustained release of IGF-1 [161]. Compared with the SF-free microsphere system, the composite hydrogel can release IGF-1 relatively slowly and continuously. Furthermore, intramuscular injection of complex hydrogels reduced infarct size and improved cardiac function after 28 d. To address the release limitation problem, a dual-function MI-responsive on-demand growth factor release system was developed to promote angiogenesis and inhibit cardiac remodeling by targeting MMP (matrix metalloproteinase)-2/9 upregulated after MI. By combining collagen amine with GSH (glutathione) sulfhydryl group, GSH-modified collagen hydrogels were prepared [162]. The recombinant protein GST-TIMP-bFGF was synthesized by the fusion of bFGF with GST (glutathione s-transferase) and MMP-2/9 shearable peptide PLGLAG (TIMP). The specific binding of GST and GSH significantly increased the loading capacity of GST-TIMP-bFGF in collagen-GSH hydrogel. During myocardial infarction, the TIMP peptide wrapped between GST and bFGF responds to MMP and was released on demand, which promoted angiogenesis and reduced cardiac remodeling through synchronous control of binding and responsive release, indicating a promising treatment strategy for ischemic heart disease.

### 5.2. For Drug Loading

#### 5.2.1. Hydrogels for Direct Loading of Drugs

Curcumin, as an effective component of Chinese traditional medicine curcumin, has a significant cardiac protective effect on reperfusion injury, inhibiting ventricular remodeling caused by pressure load or myocardial infarction and improving cardiac function. However, curcumin has poor water solubility and low bioavailability [163]. Hydrogel-loaded curcumin will significantly improve its targeting activity and promote the sustained release. For example, a curcumin carrier hydrogel reduced cardiomyocytes apoptosis and hypoxia-reoxygenation-injury-induced ROS formation by maintaining Cx43 phosphorylation [164]. This design also promoted autophagy, reduced mitochondrial damage, and activated the JAK2/STAT3 (Janus kinase 2/signal transducer and activator of transcription) pathway. Furthermore, in situ injection of curcumin and NO into hydrogel will exert a synergistic effect to promote angiogenesis [165]. As another example, natural ingredient colchicine has an anti-inflammatory effect, while its systemic toxicity and narrow therapeutic window restricted its biomedical application [166,167]. In order to solve this issue, a polymeric hydrogel loaded with colchicine was designed and showed its ability to effectively relieve heart inflammation [168], to inhibit myocardial apoptosis and fibrosis, to improve cardiac function and structure, and to increase the survival rate of mice, without severe systemic toxicity.

#### 5.2.2. Nanomaterials Packaging for Drug Loading

TIIA (Tashinone IIA), one of the main active components of *Salvia miltiorrhiza Bge*, has been widely used in the treatment of cardiovascular diseases in China. It could significantly improve the cardiac function of infarcted myocardium [169], while its poor solubility, short half-life, and low drug loading of loading materials limit its application [170,171]. However, TIIA can form TIIA nanoparticles through the hydrophobic self-assembly method. In this case, the researcher deposited PDA (pyridoxylamine) layer in situ on TIIA nanoparticles to prepare core-shell TIIA@PDA nanoparticles, which had a high drug loading capacity. On the other hand, in order to restore cardiac function after myocardial infarction [172], hyperbranched ROS-sensitive macromer HB-PBAE (hyperbranched-Poly(β-amino esters)) with polyacrylate terminal group was synthesized by dynamic controlled Michael addition method. HB-PBAE was crosslinked with HA-SH (thio-hyaluronic acid) to form in situ hydrogel. Chemical crosslinking of thiocyanate and quinone groups on PDA was performed. In this case, TIIA@PDA NPs (nanoparticles) could be captured. Injection of ROS-sensitive hydrogels enhanced by TIIA@PDA NPs into infarcted hearts reversed the harmful microenvironment and inhibited the expression of inflammatory factors, such as IL-1β (interleukin 1β), IL-6 (interleukin 6), and TNF-α (tumor necrosis factor-α), thereby restoring myocardial function.

In addition to realizing high drug load and local sustained release, nanomaterials also have application in dual drug delivery systems [173], which can realize independent release of different drugs [174]. For example, tPA (tissue plasminogen activator) is commonly used for the treatment of large and small vessel myocardial infarction. The small molecule ROCK (Rho-associated kinase) inhibitor Y-27632 could eliminate the pathological process of fibrosis [175]. A dual drug delivery system was designed using a fibrin-specific poly (N-isopropylacrylamide) nanohydrogel, composed of a core-shell colloidal hydrogel structure. The architecture consists of a highly crosslinked nanogel core and loosely crosslinked shell to control the load and release of two different MI therapies. tPA would be mainly divided into relatively loose crosslinking shells, and diffusion of Y-27632 took place through crosslinked gel core layer. This design is the pioneer report of tPA release to solve primary myocardial infarction, occlusion, and fibrin deposition issues. Furthermore, the persistently released Y-27632 could block fibrosis pathogenesis and progress of key cellular responses to prevent heart scarring [160], It also addressed the need to rebuild blood flow and inhibit myocardial fibrosis after I/R injury (Figure 5d). Inspired by Ferrero’s core-shell structure, another hydrophobic DMOG (dimethyloxy glycine) NPs as core and a water-soluble drug EGCG (epigallocatechin-3-gallate) encapsulated by dopamine were synthesized. EGCG was within a simulated nano-carrier composed of thick PDA shells formed by self-polymerization through π-π interaction, which had the triadic ability to load two different drugs [176]. In this case, an injectable hydrogel was obtained with the ability to eliminate ROS and acted as a cross-linking agent to HA-SH. Ferrero-like NPs and HA-SH could rapidly form hydrogels with stable mechanical properties, strong ROS capture ability, and programmed release of EGCG and DMOG. After four weeks of myocardial infraction in rats, ejection fraction (EF) increased by 23.7%, infarct area decreased by 21.1%, and fibrosis area decreased by 24.4%.

#### 5.2.3. Multifactorial Combination Therapy

Hydrogel-loaded multi-drug cocktail therapy for myocardial infarction is one of the ways of multi-factor combined therapy in the treatment of myocardial infarction. For example, hyaluronic acid hydrogel was designed for myocardial joint injection, with the loading of platelet-rich plasma, allopurinol, ascorbic acid, and ibuprofen. This multifactor combination strategy could effectively thicken the infarcted myocardium, improve the host cell vitality, and realize attenuation of myocardial remodeling or dysfunction after myocardial infarction in pig animal models [177]. Combined infusion control might also have synergistic effects. For example, it might potentially improve the function of damaged myocardium while alleviating adverse cardiac remodeling after acute myocardial infarction. As a typical case, AST NPs (astragaloside IV nanoparticles) were prepared by a hydrophobic self-assembly method, and the hyperbranched polymers PEGDA-PBA (polyethylene glycol diacrylate 4-vinylbenzene boronic acid) and HA-SH formed in situ hydrogel to adsorb AST NPs for drug targeting and sustained release [178]. In the meanwhile, the hydrogel was doped with GNRs (gold nanorods) to provide electrical stimulation, which significantly improved myocardial infarction-induced cardiac dysfunction and cardiac remodeling by stimulating angiogenesis, promoting cell-cell signal transduction, and inhibiting apoptosis.

### 5.3. For Loading of Exogenous Gene

#### 5.3.1. Loading of Genes and Plasmids

With the development of gene transfer technology, introducing gene therapy for cardiovascular diseases such as heart failure has been gradually arriving at the stage of clinical trials [179,180]. It is worth mentioning that non-viral transfection vectors were of satisfying stability and biological safety [181]. In the case of using hydrogels as non-viral gene delivery vectors, it might also show good biocompatibility as a gene delivery carrier and prolonged gene expression [182,183]. As a typical case, the luciferase plasmid loaded within temperature-responsive hydrogels showed a four times increase in gene expression in mice, in comparison with naked plasmids only [184]. While upon HVEGF (human vascular endothelial growth factor) plasmid could be loaded in gel formulation within the infarction area, continuous HVEGF expression might increase capillary density and vascular formation in an effective manner.

In another case, RNAi (ribonucleic acid interference) is an effective gene silencing technology, and the local targeting RNAi therapy strategy has strong specificity and high efficiency in the treatment of heart disease [185,186,187]. Previous reports have synthesized the plasmid of ACE-shRNA (angiotensin-converting enzyme-short hairpin RNA) [188]. The DNA (deoxyribonucleic acid) sequence of rat ACE-shRNA was cloned into pgenesil-1 plasmid and mixed with Dex-PCL-HEMA/PNIPAAm (dextran-poly(e-caprolactone)-2-hydroxylethylmethacrylate-poly(N-isopropylacrylamide) hydrogel [189]. The hydrogel loaded with ACE-shRNA plasmid prolongates the expression time of the gene in vivo, thus prolonging the silence time of the ACE gene and achieving greater myocardial protection. This method of silencing the ACE gene could completely block the effect of ACE after myocardial infarction compared with lowering Ang II (Human angiotension Ⅱ) level with ACE inhibitor [190]. In addition, the induction of MSCs by microRNA and cardiomyocytes combined with hydrogel can improve the differentiation efficiency of MSCs into iCMs (induced cardiomyocyte-like cells) to improve the cardiac function of the MI model after transplantation [191]. In particular, miR-21-5p (microRNA-21-5p), which is highly expressed in endothelial cells, can stimulate angiogenesis by targeting anti-angiogenic genes [192]. miR-21-5p was loaded with MSN (mesoporous silica nanoparticles) with anti-inflammatory potential, and the complex was loaded with pH-responsive hydrogel to obtain Gel@MSN/miR-21-5p. In this report, on-demand delivery was achieved using pH triggering in the acidic infarct zone, and miR-21-5p was delivered by the MSN/miR-21-5p complex in the second stage. The synergistic effect of anti-inflammatory and pro-vascular effects can effectively reduce myocardial infarct size in pigs [193], restore contractility of damaged myocardium, and significantly increase capillary density in the border zone, thereby significantly improving myocardial function [194]. In short, the gene therapy with the assistance of injectable hydrogels could also be favored.

#### 5.3.2. Combination Therapy

**Figure 6:**
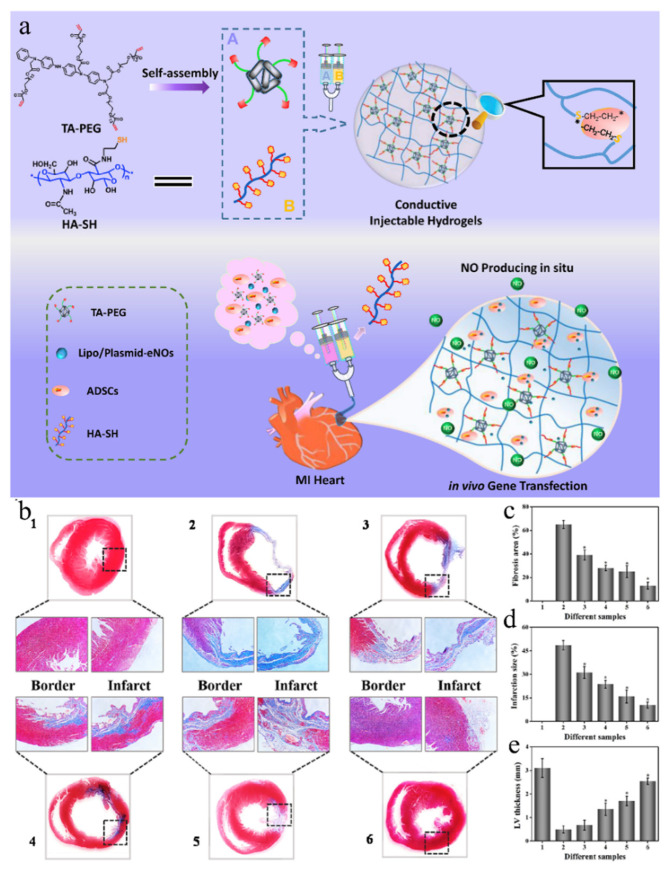
(**a**) Schematic diagram of an injectable conductive hydrogel loaded with plasmid DNA-eNOs nanoparticles and ADSCs for treatment of myocardial infarction. In this design, (1) the conductive hydrogels would enhance the electrical communications to restore the heart functions; (2) ADSCs encapsulated in hydrogels can be directly injected into the infarcted myocardium to alleviate the inflammation environment and compensate the cell loss after MI; (3) the up-regulated eNOs expression in myocardium tissue would promote neo-vascularization and enhance the mature of myocardium. (**b**) Cardiac structures in the six groups as revealed by Masson’s trichrome straining and quantitative analysis of fibrosis area (**c**), infarct size (**d**), and LV thickness (**e**). (**1**) Normal; (**2**) MI; (**3**) PEG-4A/HA-SH; (**4**) TA-PEG/HA-SH; (**5**) TA-PEG/HA-SH/ADSCs; (**6**) TA-PEG/HA-SH/ADSCs/Gene; (Reprinted with permission from Ref. [196]. 2022, Elsevier).

MI, resulting in massive myocardial cell death, adverse microenvironmental changes, loss of electrical communication in fibrotic scars, and inadequate blood supply to the infarcted myocardium, are three key aspects of MI treatment [195]. Lipid/plasmid DNA-eNOs (endothelial nitric oxide synthase) nanocomplexes were loaded into soft conductive hydrogels with equivalent myocardial conductivity and anti-fatigue properties. By co-encapsulation with ADSCs (adipose-derived stem cells) in vitro, the expression of NO was also significantly up-regulated by examining the enhanced NOx concentration in the medium after seven days, indicating gene transfection of ADSCs [196]. After the hydrogel was injected into the infarcted heart, QRT-PCR (quantitative real time polymerse chain reaction) showed that eNOs, VEGFA, Ang-1, Cx43, and Cdh-2 mRNA gene expressions were all up-regulated. The cardiac ejection fraction (EF) was significantly increased, the QRS interval was shortened, the infarct size was reduced, the fibrosis area was reduced, and the blood vessel density was increased, indicating a significant improvement in cardiac function (as shown in Figure 6).

This conceptual study of combination therapy demonstrated a holistic approach to MI that enhances electrical communication to restore cardiac function, while ADSCs injected into hydrogels of infarcted myocardium alleviate the inflammatory environment, compensate for cell loss, and upregulate NO expression in myocardial tissue, with the ability to promote neovascularization and myocardial maturation.

## 6. Conclusions and Perspective

At present, hydrogel has made breakthrough progress in the treatment of myocardial infarction. As a substitute for myocardial infarction, the material itself not only has the potential of treating myocardial infarction, but also has great development potential in effective cell encapsulation, as well as bioactive factors, proteins, drugs, or genes delivery. However, the injection volume and time of injectable hydrogel, the concentration of loading factors and drugs, the matching of degradation rate and myocardial remodeling, and the physical and biological mechanisms of hydrogel for myocardial repair need to be further clarified.

First of all, regarding the existing hydrogel treatment strategies, the injection amount of hydrogel in different treatment approaches and the injection time after MI need to be further studied, due to infarction area size differences in the clinical application. It is necessary to individualize infarction and injection amounts in order to achieve the optimal treatment effect. Furthermore, for the case of bioactive factor, drug, and gene loading, hydrogel injection time and the release of the load composition concentration should also be adapted to the corresponding pathologic stage. At present, most studies are limited to the effect of hydrogel injection at a single time on cardiac function, and there are few studies on the effect of injection time after MI. However, it has been proved that the time of hydrogel injection after myocardial infarction has a certain correlation with the efficacy [197,198], which is worth further study on this issue.

Secondly, the pathological change of myocardial infarction is a progressive process, which gradually leads to ventricular remodeling. The degradation rate of hydrogel should match that of myocardial remodeling, and the degradation rate of hydrogel should be automatically degraded with myocardial remodeling. In this case, the degraded hydrogel should have sufficient mechanical force to continue to support the myocardial tissue. Recently, the EMH (elastin simulated hydrogel) loaded with SAB-PDA (salvianolic acid and polydopamine nanoparticles) solved the problem that the mechanical strength of hydrogel structure was reduced by the continuous exercise of myocardial tissue [199]. Under the up-regulation of TGase (glutamine transaminase) in myocardial tissue after myocardial infarction, SAB-PDA/EMH with higher mechanical strength can be formed, which makes the hydrogel material have better self-healing ability. More studies are also needed on how different hydrogel materials can be degraded while maintaining their mechanical properties. In addition, it is necessary to consider that the degraded fragments of hydrogel should not cause other adverse reactions in vivo, and the massive release of cells, drugs, and factors caused by degradation may also affect the treatment efficiency or produce side effects. Therefore, we also need to establish a more complete evaluation method for the efficacy and side effects of hydrogels in the treatment of myocardial infarction [200].

Finally, MI is the physiological and pathological mechanism study upon hydrogel treatments after MI had revealed that hydrogel might reduce stress, influence the geometrical shape and thickness of the ventricular wall, and promote angiogenesis [201]. However, it is also worth mentioning that the interaction between this hydrogel formulation and the microenvironment of MI still lacks exploration [202,203].

In short, the application of hydrogel in the treatment of MI has made significant progress in clinical research [204,205]. Based on this, different treatment strategies related to hydrogel will provide a variety of treatment means and approaches to serve patients. This review summarized a number of functional hydrogels in the treatment of MI, as well as various biological therapies combining cell, factor, protein, and drug-gene therapy for MI. Injectable hydrogel as a new biological material, although there is no precise and systematic study on the efficacy of various aspects, the satisfactory progress of hydrogel formulation in the field of myocardial tissue engineering will definitely bring better therapeutic effects for patients with MI. To achieve an optimal therapeutic effect, it might need to find the optimal injection time and load component concentration in the next few decades to explore the important mechanism of hydrogel in the direction of MI. Furthermore, the intelligent release of load components based on the myocardial microenvironment and better self-healing ability of hydrogel based on the dynamic process of myocardial continuous movement and ventricular remodeling will also be the future research direction. It is one of the key points of materials science and myocardial tissue engineering research to create a “cocktail therapy” with the optimal curative effect by rationally matching the modification of hydrogel, the combination of cells, growth factors, proteins, and drugs, in order to achieve a long-term curative effect.

## References

[1] Virani S.S., Alonso A., Benjamin E.J., Bittencourt M.S., Callaway C.W., Carson A.P., Chamberlain A.M., Chang A.R., Cheng S., Delling F.N. (2020). Heart Disease and Stroke Statistics—2020 Update: A Report from the American Heart Association. Circulation.

[2] Jiang J., Guo Y., Huang Z., Zhang Y., Wu D., Liu Y. (2022). Adjacent surface trajectory planning of robot-assisted tooth preparation based on augmented reality. Eng. Sci. Technol. Int. J..

[3] Gao H., Hsu P.-H., Li K., Zhang J. (2020). The Real Effect of Smoking Bans: Evidence from Corporate Innovation. J. Financ. Quant. Anal..

[4] Antman E., Bassand J.P., Klein W., Ohman M., Lopez Sendon J.L., Rydén L., Simoons M., Tendera M. (2001). Myocardial infarction redefined—A consensus document of the Joint European Society of Cardiology American College of Cardiology Committee for the Redefinition of Myocardial Infarction: The Joint European Society of Cardiology/American College of Cardiology Committee. J. Am. Coll. Cardiol..

[5] González A., Schelbert E.B., Díez J., Butler J. (2018). Myocardial Interstitial Fibrosis in Heart Failure Biological and Translational Perspectives. J. Am. Coll. Cardiol..

[6] Ziaeian B., Fonarow G.C. (2016). Epidemiology and aetiology of heart failure. Nat. Rev. Cardiol..

[7] Mancini D., Colombo P.C. (2015). Left Ventricular Assist Devices: A Rapidly Evolving Alternative to Transplant. J. Am. Coll. Cardiol..

[8] Alkan M., Madanieh R., Shah N.N., Dogar M.U., Shah P.N., Ishtiaq S., Kosmas C.E., Vittorio T.J. (2017). Regenerative Stem Cell Therapy Optimization via Tissue Engineering in Heart Failure with Reduced Ejection Fraction. Cardiovasc. Eng. Technol..

[9] Blackburn N.J.R., Sofrenovic T., Kuraitis D., Ahmadi A., McNeill B., Deng C., Rayner K.J., Zhong Z., Ruel M., Suuronen E.J. (2015). Timing underpins the benefits associated with injectable collagen biomaterial therapy for the treatment of myocardial infarction. Biomaterials.

[10] Rocker A.J., Lee D.J., Shandas R., Park D. (2020). Injectable Polymeric Delivery System for Spatiotemporal and Sequential Release of Therapeutic Proteins to Promote Therapeutic Angiogenesis and Reduce Inflammation. ACS Biomater. Sci. Eng..

[11] Kapnisi M., Mansfield C., Marijon C., Guex A.G., Perbellini F., Bardi I., Humphrey E.J., Puetzer J.L., Mawad D., Koutsogeorgis D. (2018). Auxetic Cardiac Patches with Tunable Mechanical and Conductive Properties toward Treating Myocardial Infarction. Adv. Funct. Mater..

[12] Li Z., Teng M., Yang R., Lin F., Fu Y., Lin W., Zheng J., Zhong X., Chen X., Yang B. (2022). Sb-doped WO_3_ based QCM humidity sensor with self-recovery ability for real-time monitoring of respiration and wound. Sens. Actuators B Chem..

[13] Peña B., Laughter M., Jett S., Rowland T.J., Taylor M.R.G., Mestroni L., Park D. (2018). Injectable Hydrogels for Cardiac Tissue Engineering. Macromol. Biosci..

[14] Guan S., Li J., Zhang K., Li J. (2021). Environmentally responsive hydrogels for repair of cardiovascular tissue. Heart Fail. Rev..

[15] Diaz M.D., Tran E., Spang M., Wang R., Gaetani R., Luo C.G., Braden R., Hill R.C., Hansen K.C., DeMaria A.N. (2021). Injectable Myocardial Matrix Hydrogel Mitigates Negative Left Ventricular Remodeling in a Chronic Myocardial Infarction Model. JACC Basic Transl. Sci..

[16] Yan J., Yao Y., Yan S., Gao R., Lu W., He W. (2020). Chiral Protein Supraparticles for Tumor Suppression and Synergistic Immunotherapy: An Enabling Strategy for Bioactive Supramolecular Chirality Construction. Nano Lett..

[17] Obireddy S.R., Lai W.-F. (2021). Multi-Component Hydrogel Beads Incorporated with Reduced Graphene Oxide for pH-Responsive and Controlled Co-Delivery of Multiple Agents. Pharmaceutics.

[18] Lai W.-F., Gui D., Wong M., Döring A., Rogach A.L., He T., Wong W.-T. (2021). A self-indicating cellulose-based gel with tunable performance for bioactive agent delivery. J. Drug Deliv. Sci. Technol..

[19] Tibaut M., Mekis D., Petrovic D. (2017). Pathophysiology of Myocardial Infarction and Acute Management Strategies. Cardiovasc. Hematol. Agents Med. Chem..

[20] Cahill T.J., Choudhury R.P., Riley P.R. (2017). Heart regeneration and repair after myocardial infarction: Translational opportunities for novel therapeutics. Nat. Rev. Drug Discov..

[21] Frangogiannis N.G. (2015). Pathophysiology of Myocardial Infarction. Compr. Physiol..

[22] Montone R.A., Camilli M., Del Buono M.G., Meucci M.C., Gurgoglione F., Russo M., Crea F., Niccoli G. (2020). No-reflow: Update on diagnosis, pathophysiology and therapeutic strategies. G. Ital. Di Cardiol..

[23] Bhatt A.S., Ambrosy A.P., Velazquez E.J. (2017). Adverse Remodeling and Reverse Remodeling After Myocardial Infarction. Curr. Cardiol. Rep..

[24] Smit M., Coetzee A., Lochner A., Smit M., Coetzee A., Lochner A. (2020). The Pathophysiology of Myocardial Ischemia and Perioperative Myocardial Infarction. J. Cardiothorac. Vasc. Anesthesia.

[25] Adapala R.K., Kanugula A.K., Paruchuri S., Chilian W.M., Thodeti C.K. (2020). TRPV4 deletion protects heart from myocardial infarction-induced adverse remodeling via modulation of cardiac fibroblast differentiation. Basic Res. Cardiol..

[26] Schloss M.J., Horckmans M., Nitz K., Duchene J., Drechsler M., Bidzhekov K., Scheiermann C., Weber C., Soehnlein O., Steffens S. (2016). The time-of-day of myocardial infarction onset affects healing through oscillations in cardiac neutrophil recruitment. EMBO Mol. Med..

[27] Puhl S.-L., Steffens S. (2019). Neutrophils in Post-myocardial Infarction Inflammation: Damage vs. Resolution?. Front. Cardiovasc. Med..

[28] Zekios K.C., Mouchtouri E.T., Lekkas P., Nikas D.N., Kolettis T.M. (2021). Sympathetic Activation and Arrhythmogenesis after Myocardial Infarction: Where Do We Stand?. J. Cardiovasc. Dev. Dis..

[29] Barouti G., Liow S.S., Dou Q., Ye H., Orione C., Guillaume S.M., Loh X.J. (2016). New Linear and Star-Shaped Thermogelling Poly([*R*]-3-hydroxybutyrate) Copolymers. Chem. A Eur. J..

[30] Gan L., Deen G.R., Loh X., Gan Y. (2001). New stimuli-responsive copolymers of N-acryloyl-N′-alkyl piperazine and methyl methacrylate and their hydrogels. Polymer.

[31] Loh X.J., Cheong W.C.D., Li J., Ito Y. (2009). Novel poly(N-isopropylacrylamide)-poly[(R)-3-hydroxybutyrate]-poly(N-isopropylacrylamide) triblock copolymer surface as a culture substrate for human mesenchymal stem cells. Soft Matter.

[32] Loh X.J., Gong J., Sakuragi M., Kitajima T., Liu M., Li J., Ito Y. (2009). Surface Coating with a Thermoresponsive Copolymer for the Culture and Non-Enzymatic Recovery of Mouse Embryonic Stem Cells. Macromol. Biosci..

[33] Loh X.J., Nguyen V.P.N., Kuo N., Li J. (2011). Encapsulation of basic fibroblast growth factor in thermogelling copolymers preserves its bioactivity. J. Mater. Chem..

[34] Loh X.J., Wu Y.-L. (2015). Cationic star copolymers based on β-cyclodextrins for efficient gene delivery to mouse embryonic stem cell colonies. Chem. Commun..

[35] Loh X.J., Yee B.J.H., Chia F.S. (2012). Sustained delivery of paclitaxel using thermogelling poly(PEG/PPG/PCL urethane)s for enhanced toxicity against cancer cells. J. Biomed. Mater. Res. Part A.

[36] Loh X.J., Zhang Z.-X., Mya K.Y., Wu Y.-L., Bin He C., Li J. (2010). Efficient gene delivery with paclitaxel-loaded DNA-hybrid polyplexes based on cationic polyhedral oligomeric silsesquioxanes. J. Mater. Chem..

[37] Nguyen V.P.N., Kuo N., Loh X.J. (2011). New biocompatible thermogelling copolymers containing ethylene-butylene segments exhibiting very low gelation concentrations. Soft Matter.

[38] Dai W., Wold L.E., Dow J.S., Kloner R.A. (2005). Thickening of the Infarcted Wall by Collagen Injection Improves Left Ventricular Function in Rats: A Novel Approach to Preserve Cardiac Function After Myocardial Infarction. J. Am. Coll. Cardiol..

[39] Yu J., Christman K.L., Chin E., Sievers R.E., Saeed M., Lee R.J. (2009). Restoration of left ventricular geometry and improvement of left ventricular function in a rodent model of chronic ischemic cardiomyopathy. J. Thorac. Cardiovasc. Surg..

[40] Chen F.-M., Liu X. (2016). Advancing biomaterials of human origin for tissue engineering. Prog. Polym. Sci..

[41] Dimatteo R., Darling N.J., Segura T. (2018). In Situ forming injectable hydrogels for drug delivery and wound repair. Adv. Drug Deliv. Rev..

[42] Ifkovits J.L., Tous E., Minakawa M., Morita M., Robb J.D., Koomalsingh K.J., Gorman J.H., Gorman R.C., Burdick J.A. (2010). Injectable hydrogel properties influence infarct expansion and extent of postinfarction left ventricular remodeling in an ovine model. Proc. Natl. Acad. Sci. USA.

[43] Singelyn J.M., DeQuach J.A., Seif-Naraghi S.B., Littlefield R.B., Schup-Magoffin P.J., Christman K.L. (2009). Naturally derived myocardial matrix as an injectable scaffold for cardiac tissue engineering. Biomaterials.

[44] Seif-Naraghi S.B., Singelyn J.M., Salvatore M.A., Osborn K.G., Wang J.J., Sampat U., Kwan O.L., Strachan G.M., Wong J., Schup-Magoffin P.J. (2013). Safety and Efficacy of an Injectable Extracellular Matrix Hydrogel for Treating Myocardial Infarction. Sci. Transl. Med..

[45] Contessotto P., Orbanić D., Da Costa M., Jin C., Owens P., Chantepie S., Chinello C., Newell J., Magni F., Papy-Garcia D. (2021). Elastin-like recombinamers-based hydrogel modulates post-ischemic remodeling in a non-transmural myocardial infarction in sheep. Sci. Transl. Med..

[46] McLaughlin S., McNeill B., Podrebarac J., Hosoyama K., Sedlakova V., Cron G., Smyth D., Seymour R., Goel K., Liang W. (2019). Injectable human recombinant collagen matrices limit adverse remodeling and improve cardiac function after myocardial infarction. Nat. Commun..

[47] Pupkaite J., Sedlakova V., Cimenci C.E., Bak M., McLaughlin S., Ruel M., Alarcon E.I., Suuronen E.J. (2020). Delivering More of an Injectable Human Recombinant Collagen III Hydrogel Does Not Improve Its Therapeutic Efficacy for Treating Myocardial Infarction. ACS Biomater. Sci. Eng..

[48] Anker S.D., Coats A.J.S., Cristian G., Dragomir D., Pusineri E., Piredda M., Bettari L., Dowling R., Volterrani M., Kirwan B.-A. (2015). A prospective comparison of alginate-hydrogel with standard medical therapy to determine impact on functional capacity and clinical outcomes in patients with advanced heart failure (AUGMENT-HF trial). Eur. Heart J..

[49] Ruvinov E., Cohen S. (2016). Alginate biomaterial for the treatment of myocardial infarction: Progress, translational strategies, and clinical outlook. Adv. Drug Deliv. Rev..

[50] Li Y., Yang H.Y., Lee D.S. (2021). Advances in biodegradable and injectable hydrogels for biomedical applications. J. Control. Release.

[51] Darge H.F., Andrgie A.T., Tsai H.-C., Lai J.-Y. (2019). Polysaccharide and polypeptide based injectable thermo-sensitive hydrogels for local biomedical applications. Int. J. Biol. Macromol..

[52] Hasan A., Khattab A., Islam M.A., Hweij K.A., Zeitouny J., Waters R., Sayegh M., Hossain M., Paul A. (2015). Injectable Hydrogels for Cardiac Tissue Repair after Myocardial Infarction. Adv. Sci..

[53] Wang B., Wu C., He S., Wang Y., Wang D., Tao H., Wang C., Pang X., Li F., Yuan Y. (2022). V1-Cal hydrogelation enhances its effects on ventricular remodeling reduction and cardiac function improvement post myocardial infarction. Chem. Eng. J..

[54] Liu Z., Wang H., Wang Y., Lin Q., Yao A., Cao F., Li D., Zhou J., Duan C., Du Z. (2012). The influence of chitosan hydrogel on stem cell engraftment, survival and homing in the ischemic myocardial microenvironment. Biomaterials.

[55] Jin K., Yan Y., Chen M., Wang J., Pan X., Liu X., Liu M., Lou L., Wang Y., Ye J. (2022). Multimodal deep learning with feature level fusion for identification of choroidal neovascularization activity in age-related macular degeneration. Acta Ophthalmol..

[56] Raiter A., Bechor Z., Kleiman M., Leshem-Lev D., Battler A., Hardy B. (2010). Angiogenic Peptides Improve Blood Flow and Promote Capillary Growth in a Diabetic and Ischaemic Mouse Model. Eur. J. Vasc. Endovasc. Surg..

[57] Shu Y., Hao T., Yao F., Qian Y., Wang Y., Yang B., Li J., Wang C. (2015). RoY Peptide-Modified Chitosan-Based Hydrogel to Improve Angiogenesis and Cardiac Repair under Hypoxia. ACS Appl. Mater. Interfaces.

[58] Cui Z., Ni N.C., Wu J., Du G.-Q., He S., Yau T.M., Weisel R.D., Sung H.-W., Li R.-K. (2018). Polypyrrole-chitosan conductive biomaterial synchronizes cardiomyocyte contraction and improves myocardial electrical impulse propagation. Theranostics.

[59] Zhan J., Liao X., Fan X., Zhang J., Li H., Cai Y., Qiu X. (2022). An injectable and conductive TEMPOL/polypyrrole integrated peptide co-assembly hydrogel promotes functional maturation of cardiomyocytes for myocardial infarction repair. Compos. Part B Eng..

[60] Köhler A.C., Sag C.M., Maier L.S. (2014). Reactive oxygen species and excitation–contraction coupling in the context of cardiac pathology. J. Mol. Cell. Cardiol..

[61] Prosser B.L., Ward C.W., Lederer W.J. (2011). X-ROS Signaling: Rapid Mechano-Chemo Transduction in Heart. Science.

[62] Looi Y.H., Grieve D.J., Siva A., Walker S.J., Anilkumar N., Cave A.C., Marber M., Monaghan M.J., Shah A.M. (2008). Involvement of Nox2 NADPH Oxidase in Adverse Cardiac Remodeling after Myocardial Infarction. Hypertension.

[63] Spaulding K.A., Zhu Y., Takaba K., Ramasubramanian A., Badathala A., Haraldsson H., Collins A., Aguayo E., Shah C., Wallace A.W. (2020). Myocardial injection of a thermoresponsive hydrogel with reactive oxygen species scavenger properties improves border zone contractility. J. Biomed. Mater. Res. Part A.

[64] Wang S., Yao Y., Zhou T., Xie J., Ding J., Cao W., Shen L., Zhu Y., Gao C. (2022). Preservation of cardiac functions post myocardial infarction In Vivo by a phenylboric acid-grafted hyaluronic hydrogel with anti-oxidation and accelerated degradation under oxidative microenvironment. Compos. Part B Eng..

[65] Yellon D.M., Hausenloy D.J. (2007). Myocardial Reperfusion Injury. N. Eng. J. Med..

[66] Viola H.M., Arthur P.G., Hool L.C. (2007). Transient Exposure to Hydrogen Peroxide Causes an Increase in Mitochondria-Derived Superoxide as a Result of Sustained Alteration in L-Type Ca^2+^ Channel Function in the Absence of Apoptosis in Ventricular Myocytes. Circ. Res..

[67] Li Y., Ha T., Gao X., Kelley J., Williams D.L., Browder I.W., Kao R.L., Li C. (2004). NF-kappa B activation is required for the development of cardiac hypertrophy In Vivo. Am. J. Physiol. Heart Circ. Physiol..

[68] Zorov D.B., Filburn C.R., Klotz L.O., Zweier J.L., Sollott S.J. (2000). Reactive oxygen species (ROS)-induced ROS release: A new phenomenon accompanying induction of the mitochondrial permeability transition in cardiac myocytes. J. Exp. Med..

[69] Hardy N., Viola H.M., Johnstone V.P.A., Clemons T.D., Szappanos H.C., Singh R., Smith N.M., Iyer K.S., Hool L.C. (2015). Nanoparticle-Mediated Dual Delivery of an Antioxidant and a Peptide against the L-Type Ca^2+^ Channel Enables Simultaneous Reduction of Cardiac Ischemia-Reperfusion Injury. ACS Nano.

[70] Ferrara N., Gerber H.P., LeCouter J. (2003). The biology of VEGF and its receptors. Nat. Med..

[71] Webber M.J., Tongers J., Newcomb C.J., Marquardt K.-T., Bauersachs J., Losordo D.W., Stupp S.I. (2011). Supramolecular nanostructures that mimic VEGF as a strategy for ischemic tissue repair. Proc. Natl. Acad. Sci. USA.

[72] Yuan Z., Tsou Y.-H., Zhang X.-Q., Huang S., Yang Y., Gao M., Ho W., Zhao Q., Ye X., Xu X. (2019). Injectable Citrate-Based Hydrogel as an Angiogenic Biomaterial Improves Cardiac Repair after Myocardial Infarction. ACS Appl. Mater. Interfaces.

[73] Zhang Y., Murugesan P., Huang K., Cai H. (2020). NADPH oxidases and oxidase crosstalk in cardiovascular diseases: Novel therapeutic targets. Nat. Rev. Cardiol..

[74] Spinale F.G., Janicki J.S., Zile M. (2013). Membrane-Associated Matrix Proteolysis and Heart Failure. Circ. Res..

[75] Fan Z., Xu Z., Niu H., Gao N., Guan Y., Li C., Dang Y., Cui X., Liu X.L., Duan Y. (2018). An Injectable Oxygen Release System to Augment Cell Survival and Promote Cardiac Repair Following Myocardial Infarction. Sci. Rep..

[76] Ding J., Yao Y., Li J., Duan Y., Nakkala J.R., Feng X., Cao W., Wang Y., Hong L., Shen L. (2020). A Reactive Oxygen Species Scavenging and O_2_ Generating Injectable Hydrogel for Myocardial Infarction Treatment In Vivo. Small.

[77] Webb C.S., Bonnema D.D., Ahmed S.H., Leonardi A.H. (2006). Specific temporal profile of matrix metalloproteinase release occurs in patients after myocardial infarction—Relation to left ventricular remodeling. Circulation.

[78] Spinale F.G., Koval C.N., Deschamps A.M., Stroud R.E., Ikonomidis J.S. (2008). Dynamic Changes in Matrix Metalloprotienase Activity within the Human Myocardial Interstitium during Myocardial Arrest and Reperfusion. Circulation.

[79] Spinale F.G. (2007). Myocardial Matrix Remodeling and the Matrix Metalloproteinases: Influence on Cardiac Form and Function. Physiol. Rev..

[80] Eckhouse S.R., Purcell B.P., McGarvey J.R., Lobb D., Logdon C.B., Doviak H., O’Neill J.W., Shuman J.A., Novack C.P., Zellars K.N. (2014). Local Hydrogel Release of Recombinant TIMP-3 Attenuates Adverse Left Ventricular Remodeling after Experimental Myocardial Infarction. Sci. Transl. Med..

[81] Purcell B.P., Barlow S.C., Perreault P.E., Freeburg L., Doviak H., Jacobs J., Hoenes A., Zellars K.N., Khakoo A.Y., Lee T. (2018). Delivery of a matrix metalloproteinase-responsive hydrogel releasing TIMP-3 after myocardial infarction: Effects on left ventricular remodeling. Am. J. Physiol. Heart Circ. Physiol..

[82] Mihic A., Cui Z., Wu J., Vlacic G., Miyagi Y., Li S.-H., Lu S., Sung H.-W., Weisel R.D., Li R.-K. (2015). A Conductive Polymer Hydrogel Supports Cell Electrical Signaling and Improves Cardiac Function after Implantation into Myocardial Infarct. Circulation.

[83] Zhang C., Hsieh M.-H., Wu S.-Y., Li S.-H., Wu J., Liu S.-M., Wei H.-J., Weisel R.D., Sung H.-W., Li R.-K. (2020). A self-doping conductive polymer hydrogel that can restore electrical impulse propagation at myocardial infarct to prevent cardiac arrhythmia and preserve ventricular function. Biomaterials.

[84] Zhou J., Yang X., Liu W., Wang C., Shen Y., Zhang F., Zhu H., Sun H., Chen J., Lam J. (2018). Injectable OPF/graphene oxide hydrogels provide mechanical support and enhance cell electrical signaling after implantation into myocardial infarct. Theranostics.

[85] Yang B., Yao F., Hao T., Fang W., Ye L., Zhang Y., Wang Y., Li J., Wang C. (2016). Development of Electrically Conductive Double-Network Hydrogels via One-Step Facile Strategy for Cardiac Tissue Engineering. Adv. Health Mater..

[86] Song X., Wang X., Zhang J., Shen S., Yin W., Ye G., Wang L., Hou H., Qiu X. (2021). A tunable self-healing ionic hydrogel with microscopic homogeneous conductivity as a cardiac patch for myocardial infarction repair. Biomaterials.

[87] Bao R., Tan B., Liang S., Zhang N., Wang W., Liu W. (2017). A π-π conjugation-containing soft and conductive injectable polymer hydrogel highly efficiently rebuilds cardiac function after myocardial infarction. Biomaterials.

[88] Liu Y., Guo R., Wu T., Lyu Y., Xiao M., He B., Fan G., Yang J., Liu W. (2021). One zwitterionic injectable hydrogel with ion conductivity enables efficient restoration of cardiac function after myocardial infarction. Chem. Eng. J..

[89] Sepantafar M., Maheronnaghsh R., Mohammadi H., Rajabi-Zeleti S., Annabi N., Aghdami N., Baharvand H. (2016). Stem cells and injectable hydrogels: Synergistic therapeutics in myocardial repair. Biotechnol. Adv..

[90] Yin S., Cao Y. (2021). Hydrogels for Large-Scale Expansion of Stem Cells. Acta Biomater..

[91] Don C.W., Murry C.E. (2013). Improving survival and efficacy of pluripotent stem cell–derived cardiac grafts. J. Cell. Mol. Med..

[92] Si R., Gao C., Guo R., Lin C., Li J., Guo W. (2020). Human mesenchymal stem cells encapsulated-coacervated photoluminescent nanodots layered bioactive chitosan/collagen hydrogel matrices to indorse cardiac healing after acute myocardial infarction. J. Photochem. Photobiol. B Biol..

[93] Nelson T.J., Martinez-Fernandez A., Terzic A. (2010). Induced pluripotent stem cells: Developmental biology to regenerative medicine. Nat. Rev. Cardiol..

[94] Poorna M., Jayakumar R., Chen J.-P., Mony U. (2021). Hydrogels: A potential platform for induced pluripotent stem cell culture and differentiation. Colloids Surf. B Biointerfaces.

[95] Sanganalmath S.K., Bolli R. (2013). Cell Therapy for Heart Failure A Comprehensive Overview of Experimental and Clinical Studies, Current Challenges, and Future Directions. Circ. Res..

[96] Roshanbinfar K., Esser T.U., Engel F.B. (2021). Stem Cells and Their Cardiac Derivatives for Cardiac Tissue Engineering and Regenerative Medicine. Antioxid. Redox Signal..

[97] Li X.Y., Wang T., Jiang X.J., Lin T., Ren S. 3-Dimension (3-D) Culture of Endothelial Cells In Vitro. Proceedings of the 2009 3rd International Conference on Bioinformatics and Biomedical Engineering.

[98] Christman K.L., Vardanian A.J., Fang Q., Sievers R.E., Fok H.H., Lee R.J. (2004). Injectable Fibrin Scaffold Improves Cell Transplant Survival, Reduces Infarct Expansion, and Induces Neovasculature Formation in Ischemic Myocardium. J. Am. Coll. Cardiol..

[99] Lin Y.-D., Yeh M.-L., Yang Y.-J., Tsai D.-C., Chu T.-Y., Shih Y.-Y., Chang M.-Y., Liu Y.-W., Tang A.C., Chen T.-Y. (2010). Intramyocardial Peptide Nanofiber Injection Improves Postinfarction Ventricular Remodeling and Efficacy of Bone Marrow Cell Therapy in Pigs. Circulation.

[100] Bonafè F., Govoni M., Giordano E., Caldarera C.M., Guarnieri C., Muscari C. (2014). Hyaluronan and cardiac regeneration. J. Biomed. Sci..

[101] Zhu Y., Matsumura Y., Velayutham M., Foley L.M., Hitchens T.K., Wagner W.R. (2018). Reactive oxygen species scavenging with a biodegradable, thermally responsive hydrogel compatible with soft tissue injection. Biomaterials.

[102] Rane A.A., Christman K.L. (2011). Biomaterials for the Treatment of Myocardial Infarction: A 5-Year Update. J. Am. Coll. Cardiol..

[103] Demirbilek M.E., Demirbilek M., Karahaliloğlu Z., Erdal E., Vural T., Yalçın E., Sağlam N., Denkbaş E.B. (2011). Oxidative Stress Parameters of L929 Cells Cultured on Plasma-Modified PDLLA Scaffolds. Appl. Biochem. Biotechnol..

[104] Li J., Shu Y., Hao T., Wang Y., Qian Y., Duan C., Sun H., Lin Q., Wang C. (2013). A chitosan–glutathione based injectable hydrogel for suppression of oxidative stress damage in cardiomyocytes. Biomaterials.

[105] Hao T., Li J., Yao F., Dong D., Wang Y., Yang B., Wang C. (2017). Injectable Fullerenol/Alginate Hydrogel for Suppression of Oxidative Stress Damage in Brown Adipose-Derived Stem Cells and Cardiac Repair. ACS Nano.

[106] Ding H., Ding J., Liu Q., Lin J., He M., Wu X., Chen X., Xiao C., Ren T., Zhu Y. (2022). Mesenchymal stem cells encapsulated in a reactive oxygen species-scavenging and O_2_-generating injectable hydrogel for myocardial infarction treatment. Chem. Eng. J..

[107] Zhu K., Jiang D., Wang K., Zheng D., Zhu Z., Shao F., Qian R., Lan X., Qin C. (2022). Conductive nanocomposite hydrogel and mesenchymal stem cells for the treatment of myocardial infarction and non-invasive monitoring via PET/CT. J. NanoBiotechnol..

[108] Yuan Z., Qin Q., Yuan M., Wang H., Li R. (2020). Development and novel design of clustery graphene oxide formed Conductive Silk hydrogel cell vesicle to repair and routine care of myocardial infarction: Investigation of its biological activity for cell delivery applications. J. Drug Deliv. Sci. Technol..

[109] Guilak F., Cohen D.M., Estes B.T., Gimble J.M., Liedtke W., Chen C.S. (2009). Control of stem cell fate by physical interactions with the extracellular matrix. Cell Stem Cell.

[110] Zhao X., Cui K., Li Z. (2019). The role of biomaterials in stem cell-based regenerative medicine. Futur. Med. Chem..

[111] Yao Y., Yang L., Feng L.-F., Yue Z.-W., Zhao N.-H., Li Z., He Z.-X. (2020). IGF-1C domain–modified hydrogel enhanced the efficacy of stem cells in the treatment of AMI. Stem Cell Res. Ther..

[112] Ban K., Park H.-J., Kim S., Andukuri A., Cho K.-W., Hwang J.W., Cha H.J., Kim S.Y., Kim W.-S., Jun H.-W. (2014). Cell Therapy with Embryonic Stem Cell-Derived Cardiomyocytes Encapsulated in Injectable Nanomatrix Gel Enhances Cell Engraftment and Promotes Cardiac Repair. ACS Nano.

[113] Li H., Gao J., Shang Y., Hua Y., Ye M., Yang Z., Ou C.W., Chen M. (2018). Folic Acid Derived Hydrogel Enhances the Survival and Promotes Therapeutic Efficacy of iPS Cells for Acute Myocardial Infarction. ACS Appl. Mater. Interfaces.

[114] Chen Y.-S., Tsou P.-C., Lo J.-M., Tsai H.-C., Wang Y.-Z., Hsiue G.-H. (2013). Poly(N-isopropylacrylamide) hydrogels with interpenetrating multiwalled carbon nanotubes for cell sheet engineering. Biomaterials.

[115] Li X., Zhou J., Liu Z., Chen J., Lü S., Sun H., Li J., Lin Q., Yang B., Duan C. (2014). A PNIPAAm-based thermosensitive hydrogel containing SWCNTs for stem cell transplantation in myocardial repair. Biomaterials.

[116] Zhu S., Yu C., Liu N., Zhao M., Chen Z., Liu J., Li G., Huang H., Guo H., Sun T. (2022). Injectable conductive gelatin methacrylate/oxidized dextran hydrogel encapsulating umbilical cord mesenchymal stem cells for myocardial infarction treatment. Bioact. Mater..

[117] Purcell B.P., Elser J.A., Mu A., Margulies K.B., Burdick J.A. (2012). Synergistic effects of SDF-1α chemokine and hyaluronic acid release from degradable hydrogels on directing bone marrow derived cell homing to the myocardium. Biomaterials.

[118] Li J., Lv Y., Zhu D., Mei X., Huang K., Wang X., Li Z., Zhang S., Hu S., Popowski K.D. (2022). Intrapericardial hydrogel injection generates high cell retention and augments therapeutic effects of mesenchymal stem cells in myocardial infarction. Chem. Eng. J..

[119] Li X., Tamama K., Xie X., Guan J. (2016). Improving Cell Engraftment in Cardiac Stem Cell Therapy. Stem Cells Int..

[120] Li Z., Guo X., Guan J. (2012). An oxygen release system to augment cardiac progenitor cell survival and differentiation under hypoxic condition. Biomaterials.

[121] Alemdar N., Leijten J., Camci-Unal G., Hjortnaes J., Ribas J., Paul A., Mostafalu P., Gaharwar A.K., Qiu Y., Sonkusale S. (2017). Oxygen-Generating Photo-Cross-Linkable Hydrogels Support Cardiac Progenitor Cell Survival by Reducing Hypoxia-Induced Necrosis. ACS Biomater. Sci. Eng..

[122] Niu H., Li C., Guan Y., Dang Y., Li X., Fan Z., Shen J., Ma L., Guan J. (2020). High oxygen preservation hydrogels to augment cell survival under hypoxic condition. Acta Biomater..

[123] Rota M., Padin-Iruegas M.E., Misao Y., De Angelis A., Maestroni S., Ferreira-Martins J., Fiumana E., Rastaldo R., Arcarese M.L., Mitchell T.S. (2008). Local Activation or Implantation of Cardiac Progenitor Cells Rescues Scarred Infarcted Myocardium Improving Cardiac Function. Circ. Res..

[124] Padin-Iruegas M.E., Misao Y., Davis M.E., Segers V.F., Esposito G., Tokunou T., Urbanek K., Hosoda T., Rota M., Anversa P. (2009). Cardiac Progenitor Cells and Biotinylated Insulin-Like Growth Factor-1 Nanofibers Improve Endogenous and Exogenous Myocardial Regeneration after Infarction. Circulation.

[125] Burgess K., Frati C., Meade K., Gao J., Diaz L.C., Madeddu D., Graiani G., Cavalli S., Miller A., Oceandy D. (2021). Functionalised peptide hydrogel for the delivery of cardiac progenitor cells. Mater. Sci. Eng. C.

[126] Lyu Y., Xie J., Liu Y., Xiao M., Li Y., Yang J., Yang J., Liu W. (2020). Injectable Hyaluronic Acid Hydrogel Loaded with Functionalized Human Mesenchymal Stem Cell Aggregates for Repairing Infarcted Myocardium. ACS Biomater. Sci. Eng..

[127] Wu Z., Chen G., Zhang J., Hua Y., Li J., Liu B., Huang A., Li H., Chen M., Ou C. (2017). Treatment of Myocardial Infarction with Gene-modified Mesenchymal Stem Cells in a Small Molecular Hydrogel. Sci. Rep..

[128] Chan S.S.-K., Li H.-J., Hsueh Y.-C., Lee D.S., Chen J.-H., Hwang S.-M., Chen C.-Y., Shih E., Hsieh P.C.H. (2010). Fibroblast Growth Factor-10 Promotes Cardiomyocyte Differentiation from Embryonic and Induced Pluripotent Stem Cells. PLoS ONE.

[129] Yao X., Liu Y., Gao J., Yang L., Mao D., Stefanitsch C., Li Y., Zhang J., Ou L., Kong D. (2015). Nitric oxide releasing hydrogel enhances the therapeutic efficacy of mesenchymal stem cells for myocardial infarction. Biomaterials.

[130] Riaud M., Martinez M.C., Montero-Menei C.N. (2020). Scaffolds and Extracellular Vesicles as a Promising Approach for Cardiac Regeneration after Myocardial Infarction. Pharmaceutics.

[131] Khan M., Nickoloff E., Abramova T., Johnson J., Verma S.K., Krishnamurthy P., Mackie A.R., Vaughan E., Garikipati V.N.S., Benedict C. (2015). Embryonic Stem Cell–Derived Exosomes Promote Endogenous Repair Mechanisms and Enhance Cardiac Function Following Myocardial Infarction. Circ. Res..

[132] Mateescu B., Kowal E.J.K., Van Balkom B.W.M., Bartel S., Bhattacharyya S.N., Buzás E.I., Buck A.H., de Candia P., Chow F.W.N., Das S. (2017). Obstacles and opportunities in the functional analysis of extracellular vesicle RNA—An ISEV position paper. J. Extracell. Vesicles.

[133] Han C., Zhou J., Liu B., Liang C., Pan X., Zhang Y., Zhang Y., Wang Y., Shao L., Zhu B. (2019). Delivery of miR-675 by stem cell-derived exosomes encapsulated in silk fibroin hydrogel prevents aging-induced vascular dysfunction in mouse hindlimb. Mater. Sci. Eng. C.

[134] Zhang L.-L., Xiong Y.-Y., Yang Y.-J. (2021). The Vital Roles of Mesenchymal Stem Cells and the Derived Extracellular Vesicles in Promoting Angiogenesis after Acute Myocardial Infarction. Stem Cells Dev..

[135] Sánchez-Alonso S., Alcaraz-Serna A., Sánchez-Madrid F., Alfranca A. (2018). Extracellular Vesicle-Mediated Immune Regulation of Tissue Remodeling and Angiogenesis After Myocardial Infarction. Front. Immunol..

[136] De Toro J., Herschlik L., Waldner C., Mongini C. (2015). Emerging roles of exosomes in normal and pathological conditions: New insights for diagnosis and therapeutic applications. Front. Immunol..

[137] Liu H., Gao W., Yuan J., Wu C., Yao K., Zhang L., Ma L., Zhu J., Zou Y., Ge J. (2016). Exosomes derived from dendritic cells improve cardiac function via activation of CD4^+^ T lymphocytes after myocardial infarction. J. Mol. Cell. Cardiol..

[138] Zhang Y., Cai Z., Shen Y., Lu Q., Gao W., Zhong X., Yao K., Yuan J., Liu H. (2021). Hydrogel-load exosomes derived from dendritic cells improve cardiac function via Treg cells and the polarization of macrophages following myocardial infarction. J. Nanobiotechnol..

[139] Zou Y., Li L., Li Y., Chen S., Xie X., Jin X., Wang X., Ma C., Fan G., Wang W. (2021). Restoring Cardiac Functions after Myocardial Infarction–Ischemia/Reperfusion via an Exosome Anchoring Conductive Hydrogel. ACS Appl. Mater. Interfaces.

[140] Lyu Y., Azevedo H. (2021). Supramolecular Hydrogels for Protein Delivery in Tissue Engineering. Molecules.

[141] Mathew A.P., Uthaman S., Cho K.-H., Cho C.-S., Park I.-K. (2018). Injectable hydrogels for delivering biotherapeutic molecules. Int. J. Biol. Macromol..

[142] Cimenci C.E., Blackburn N.J.R., Sedlakova V., Pupkaite J., Munoz M., Rotstein B.H., Spiegel D.A., Alarcon E.I., Suuronen E.J. (2022). Combined Methylglyoxal Scavenger and Collagen Hydrogel Therapy Prevents Adverse Remodeling and Improves Cardiac Function Post-Myocardial Infarction. Adv. Funct. Mater..

[143] Huang K., Hu S., Cheng K. (2019). A New Era of Cardiac Cell Therapy: Opportunities and Challenges. Adv. Health Mater..

[144] Tang J.M., Wang J.N., Zhang L., Zheng F., Yang J.Y., Kong X., Guo L.Y., Chen L., Huang Y.Z., Wan Y. (2011). VEGF/SDF-1 promotes cardiac stem cell mobilization and myocardial repair in the infarcted heart. Cardiovasc. Res..

[145] Windmolders S., De Boeck A., Koninckx R., Daniëls A., De Wever O., Bracke M., Hendrikx M., Hensen K., Rummens J.-L. (2014). Mesenchymal stem cell secreted platelet derived growth factor exerts a pro-migratory effect on resident Cardiac Atrial appendage Stem Cells. J. Mol. Cell. Cardiol..

[146] Virag J.A., Rolle M.L., Reece J., Hardouin S., Feigl E.O., Murry C.E. (2007). Fibroblast growth factor-2 regulates myocardial infarct repair—Effects on cell proliferation, scar contraction, and ventricular function. Am. J. Pathol..

[147] Li Z., Zhu D., Hui Q., Bi J., Yu B., Huang Z., Hu S., Wang Z., Caranasos T., Rossi J. (2021). Injection of ROS-Responsive Hydrogel Loaded with Basic Fibroblast Growth Factor into the Pericardial Cavity for Heart Repair. Adv. Funct. Mater..

[148] Carlson T.R., Feng Y., Maisonpierre P.C., Mrksich M., Morla A.O. (2001). Direct Cell Adhesion to the Angiopoietins Mediated by Integrins. J. Biol. Chem..

[149] Yang W., Liu W., Li X., Yan J., He W. (2022). Turning chiral peptides into a racemic supraparticle to induce the self-degradation of MDM. J. Adv. Res..

[150] Rask F., Mihic A., Reis L., Dallabrida S.M., Ismail N.S., Sider K., Simmons C.A., Rupnick M.A., Weisel R.D., Li R.-K. (2010). Hydrogels modified with QHREDGS peptide support cardiomyocyte survival In Vitro and after sub-cutaneous implantation. Soft Matter.

[151] Reis L.A., Chiu L.L., Wu J., Feric N., Laschinger C., Momen A., Li R.K., Radisic M. (2015). Hydrogels With Integrin-Binding Angiopoietin-1-Derived Peptide, QHREDGS, for Treatment of Acute Myocardial Infarction. Circ.Heart Fail..

[152] Epstein S.E., Kornowski R., Fuchs S., Dvorak H.F. (2001). Angiogenesis therapy—Amidst the hype, the neglected potential for serious side effects. Circulation.

[153] Yancopoulos G.D., Davis S., Gale N.W., Rudge J.S., Wiegand S.J., Holash J. (2000). Vascular-specific growth factors and blood vessel formation. Nature.

[154] Betsholtz C. (2004). Insight into the physiological functions of PDGF through genetic studies in mice. Cytokine Growth Factor Rev..

[155] Hao X., Silva E.A., Månsson-Broberg A., Grinnemo K.-H., Siddiqui A.J., Dellgren G., Wärdell E., Brodin L.Å., Mooney D.J., Sylvén C. (2007). Angiogenic effects of sequential release of VEGF-A165 and PDGF-BB with alginate hydrogels after myocardial infarction. Cardiovasc. Res..

[156] Awada H.K., Johnson N.R., Wang Y. (2015). Sequential delivery of angiogenic growth factors improves revascularization and heart function after myocardial infarction. J. Control. Release.

[157] Steele A.N., Paulsen M.J., Wang H., Stapleton L.M., Lucian H.J., Eskandari A., Hironaka C.E., Farry J.M., Baker S.W., Thakore A.D. (2020). Multi-phase catheter-injectable hydrogel enables dual-stage protein-engineered cytokine release to mitigate adverse left ventricular remodeling following myocardial infarction in a small animal model and a large animal model. Cytokine.

[158] Morine K.J., Qiao X., York S., Natov P.S., Paruchuri V., Zhang Y., Aronovitz M.J., Karas R.H., Kapur N.K. (2018). Bone Morphogenetic Protein 9 Reduces Cardiac Fibrosis and Improves Cardiac Function in Heart Failure. Circulation.

[159] Wu Y., Chang T., Chen W., Wang X., Li J., Chen Y., Yu Y., Shen Z., Yu Q., Zhang Y. (2021). Release of VEGF and BMP9 from injectable alginate based composite hydrogel for treatment of myocardial infarction. Bioact. Mater..

[160] Mihalko E., Huang K., Sproul E., Cheng K., Brown A.C. (2018). Targeted Treatment of Ischemic and Fibrotic Complications of Myocardial Infarction Using a Dual-Delivery Microgel Therapeutic. ACS Nano.

[161] Feng J., Wu Y., Chen W., Li J., Wang X., Chen Y., Yu Y., Shen Z., Zhang Y. (2020). Sustained release of bioactive IGF-1 from a silk fibroin microsphere-based injectable alginate hydrogel for the treatment of myocardial infarction. J. Mater. Chem. B.

[162] Fan C., Shi J., Zhuang Y., Zhang L., Huang L., Yang W., Chen B., Chen Y., Xiao Z., Shen H. (2019). Myocardial-Infarction-Responsive Smart Hydrogels Targeting Matrix Metalloproteinase for On-Demand Growth Factor Delivery. Adv. Mater..

[163] Jin-Rong P., Zhi-Yong Q. (2014). Drug delivery systems for overcoming the bioavailability of curcumin: Not only the nanoparticle matters. J. Nanomed..

[164] Liu J., Liu J., Xu H., Zhang Y., Chu L., Liu Q., Song N., Yang C. (2014). Novel tumor-targeting, self-assembling peptide nanofiber as a carrier for effective curcumin delivery. J. Int. J. Nanomed..

[165] Chen G., Li J., Song M., Wu Z., Zhang W., Wang Z., Gao J., Yang Z., Ou C. (2017). A Mixed Component Supramolecular Hydrogel to Improve Mice Cardiac Function and Alleviate Ventricular Remodeling after Acute Myocardial Infarction. Adv. Funct. Mater..

[166] Finkelstein Y., Aks S.E., Hutson J.R., Juurlink D.N., Nguyen P., Dubnov-Raz G., Pollak U., Koren G., Bentur Y. (2010). Colchicine poisoning: The dark side of an ancient drug. Clin. Toxicol..

[167] Mullins M., Cannarozzi A.A., Bailey T.C., Ranganathan P. (2011). Unrecognized fatalities related to colchicine in hospitalized patients. Clin. Toxicol..

[168] Chen Y., Shi J., Zhang Y., Miao J., Zhao Z., Jin X., Liu L., Yu L., Shen C., Ding J. (2020). An injectable thermosensitive hydrogel loaded with an ancient natural drug colchicine for myocardial repair after infarction. J. Mater. Chem. B.

[169] Xu W., Yang J., Wu L.-M. (2009). Cardioprotective effects of tanshinone IIA on myocardial ischemia injury in rats. Die Pharm..

[170] Zhang J., Li Y., Fang X., Zhou D., Wang Y., Chen M. (2014). TPGS-g-PLGA/Pluronic F68 mixed micelles for tanshinone IIA delivery in cancer therapy. Int. J. Pharm..

[171] Qiu S., Granet R., Mbakidi J.-P., Brégier F., Pouget C., Micallef L., Sothea-Ouk T., Leger D.Y., Liagre B., Chaleix V. (2016). Delivery of tanshinone IIA and α-mangostin from gold/PEI/cyclodextrin nanoparticle platform designed for prostate cancer chemotherapy. Bioorganic Med. Chem. Lett..

[172] Wang W., Chen J., Li M., Jia H., Han X., Zhang J., Zou Y., Tan B., Liang W., Shang Y. (2019). Rebuilding Postinfarcted Cardiac Functions by Injecting TIIA@PDA Nanoparticle-Cross-linked ROS-Sensitive Hydrogels. ACS Appl. Mater. Interfaces.

[173] Nielsen P.H., Maeng M., Busk M., Mortensen L.S., Kristensen S.D., Nielsen T.T., Andersen H.R. (2010). Primary Angioplasty Versus Fibrinolysis in Acute Myocardial Infarction. Circulation.

[174] Fang J., Koh J., Fang Q., Qiu H., Archang M.M., Hasani-Sadrabadi M.M., Miwa H., Zhong X., Sievers R., Gao D. (2020). Injectable Drug-Releasing Microporous Annealed Particle Scaffolds for Treating Myocardial Infarction. Adv. Funct. Mater..

[175] Brown A.C., Fiore V.F., Sulchek T.A., Barker T.H. (2013). Physical and chemical microenvironmental cues orthogonally control the degree and duration of fibrosis-associated epithelial-to-mesenchymal transitions. J. Pathol..

[176] Han X., Li L., Xie T., Chen S., Zou Y., Jin X., Li S., Wang M., Han N., Fan G. (2020). “Ferrero-like” nanoparticles knotted injectable hydrogels to initially scavenge ROS and lastingly promote vascularization in infarcted hearts. Sci. China Technol. Sci..

[177] Vu T.D., Pal S.N., Ti L.K., Martinez E.C., Rufaihah A.J., Ling L.H., Lee C.N., Richards A.M., Kofidis T. (2015). An autologous platelet-rich plasma hydrogel compound restores left ventricular structure, function and ameliorates adverse remodeling in a minimally invasive large animal myocardial restoration model: A translational approach: Vu and Pal “Myocardial Repair: PRP, Hydrogel and Supplements”. Biomaterials.

[178] Chen J., Han X., Deng J., Zhang J., Li L., Ni J., Huang Y., Xie X., Chen S., Ke L. (2021). An injectable hydrogel based on phenylboronic acid hyperbranched macromer encapsulating gold nanorods and Astragaloside IV nanodrug for myocardial infarction. Chem. Eng. J..

[179] Lipskaia L., Ly H., Kawase Y., Hajjar R.J., Lompre A.-M. (2007). Treatment of heart failure by calcium cycling gene therapy. Futur. Cardiol..

[180] Greenberg B., Butler J., Felker G.M., Ponikowski P., Voors A.A., Desai A.S., Barnard D., Bouchard A., Jaski B., Lyon A.R. (2016). Calcium upregulation by percutaneous administration of gene therapy in patients with cardiac disease (CUPID 2): A randomised, multinational, double-blind, placebo-controlled, phase 2b trial. Lancet.

[181] Kay M.A. (2011). State-of-the-art gene-based therapies: The road ahead. Nat. Rev. Genet..

[182] De Laporte L., Shea L.D. (2007). Matrices and scaffolds for DNA delivery in tissue engineering. Adv. Drug Deliv. Rev..

[183] Drury J.L., Mooney D.J. (2003). Hydrogels for tissue engineering: Scaffold design variables and applications. Biomaterials.

[184] Kwon J.S., Park I.K., Cho A.S., Shin S.M., Hong M.H., Jeong S.Y., Kim Y.S., Min J.-J., Jeong M.H., Kim W.J. (2009). Enhanced angiogenesis mediated by vascular endothelial growth factor plasmid-loaded thermo-responsive amphiphilic polymer in a rat myocardial infarction model. J. Control. Release.

[185] Aigner A. (2006). Delivery Systems for the Direct Application of siRNAs to Induce RNA Interference (RNAi) In Vivo. J. Biomed. Biotechnol..

[186] Zou Y., Wu H., Guo X., Peng L., Ding Y., Tang J., Guo F. (2021). MK-FSVM-SVDD: A Multiple Kernel-based Fuzzy SVM Model for Predicting DNA-binding Proteins via Support Vector Data Description. Curr. Bioinform..

[187] Zhuo Z., Wan Y., Guan D., Ni S., Wang L., Zhang Z., Liu J., Liang C., Yu Y., Lu A. (2020). A Loop-Based and AGO-Incorporated Virtual Screening Model Targeting AGO-Mediated miRNA–mRNA Interactions for Drug Discovery to Rescue Bone Phenotype in Genetically Modified Mice. Adv. Sci..

[188] Suckau L., Fechner H., Chemaly E., Krohn S., Hadri L., Kockskämper J., Westermann D., Bisping E., Ly H., Wang X. (2009). Long-term cardiac-targeted RNA interference for the treatment of heart failure restores cardiac function and reduces pathological hypertrophy. Circulation.

[189] Wan W.-G., Jiang X.-J., Li X.-Y., Zhang C., Yi X., Ren S., Zhang X.-Z. (2014). Enhanced cardioprotective effects mediated by plasmid containing the short-hairpin RNA of angiotensin converting enzyme with a biodegradable hydrogel after myocardial infarction. J. Biomed. Mater. Res. Part A.

[190] Costerousse O., Allegrini J., Clozel J.P., Ménard J., Alhenc-Gelas F. (1998). Angiotensin I-converting enzyme inhibition but not angiotensin II suppression alters angiotensin I-converting enzyme gene expression in vessels and epithelia. J. Pharmacol. Exp. Ther..

[191] Khazaei S., Soleimani M., Tafti S.H., Aghdam R.M., Hojati Z. (2021). Improvement of Heart Function after Transplantation of Encapsulated Stem Cells Induced with miR-1/Myocd in Myocardial Infarction Model of Rat. Cell Transplant..

[192] Yang H., Qin X., Wang H., Zhao X., Liu Y., Wo H.-T., Liu C., Nishiga M., Chen H., Ge J. (2019). An In Vivo miRNA Delivery System for Restoring Infarcted Myocardium. ACS Nano.

[193] Li Y., Chen X., Jin R., Chen L., Dang M., Cao H., Dong Y., Cai B., Bai G., Gooding J.J. (2021). Injectable hydrogel with MSNs/microRNA-21-5p delivery enables both immunomodification and enhanced angiogenesis for myocardial infarction therapy in pigs. Sci. Adv..

[194] Wang L.L., Liu Y., Chung J.J., Wang T., Gaffey A.C., Lu M., Cavanaugh C.A., Zhou S., Kanade R., Atluri P. (2017). Sustained miRNA delivery from an injectable hydrogel promotes cardiomyocyte proliferation and functional regeneration after ischaemic injury. Nat. Biomed. Eng..

[195] Pascual-Gil S., Garbayo E., Díaz-Herráez P., Prosper F., Blanco-Prieto M. (2015). Heart regeneration after myocardial infarction using synthetic biomaterials. J. Control. Release.

[196] Wang W., Tan B., Chen J., Bao R., Zhang X., Liang S., Shang Y., Liang W., Cui Y., Fan G. (2018). An injectable conductive hydrogel encapsulating plasmid DNA-eNOs and ADSCs for treating myocardial infarction. Biomaterials.

[197] Landa N., Miller L., Feinberg M.S., Holbova R., Shachar M., Freeman I., Cohen S., Leor J. (2008). Effect of Injectable Alginate Implant on Cardiac Remodeling and Function after Recent and Old Infarcts in Rat. Circulation.

[198] Kadner K., Dobner S., Franz T., Bezuidenhout D., Sirry M.S., Zilla P., Davies N.H. (2012). The beneficial effects of deferred delivery on the efficiency of hydrogel therapy post myocardial infarction. Biomaterials.

[199] Chen R., Zhu C., Xu L., Gu Y., Ren S., Bai H., Zhou Q., Liu X., Lu S., Bi X. (2021). An injectable peptide hydrogel with excellent self-healing ability to continuously release salvianolic acid B for myocardial infarction. Biomaterials.

[200] Midgett D., Thorn S., Ahn S., Uman S., Avendano R., Melvinsdottir I., Lysyy T., Kim J., Duncan J., Humphrey J. (2022). CineCT platform for in vivo and ex vivo measurement of 3D high resolution Lagrangian strains in the left ventricle following myocardial infarction and intramyocardial delivery of theranostic hydrogel. J. Mol. Cell. Cardiol..

[201] Kichula E.T., Wang H., Dorsey S.M., Szczesny S.E., Elliott D.M., Burdick J.A., Wenk J.F. (2014). Experimental and computational investigation of altered mechanical properties in myocardium after hydrogel injection. Ann. Biomed. Eng..

[202] Boopathy A.V., Martinez M.D., Smith A.W., Brown M.E., García A.J., Davis M.E. (2015). Intramyocardial Delivery of *Notch* Ligand-Containing Hydrogels Improves Cardiac Function and Angiogenesis Following Infarction. Tissue Eng. Part A.

[203] Chen C.-H., Chang M.-Y., Wang S.-S., Hsieh P.C.H. (2014). Injection of autologous bone marrow cells in hyaluronan hydrogel improves cardiac performance after infarction in pigs. Am. J. Physiol. Circ. Physiol..

[204] Lee L.C., Zhihong Z., Hinson A., Guccione J.M. (2013). Reduction in Left Ventricular Wall Stress and Improvement in Function in Failing Hearts using Algisyl-LVR. J. Vis. Exp. JoVE.

[205] Traverse J.H., Henry T.D., Dib N., Patel A.N., Pepine C., Schaer G.L., DeQuach J.A., Kinsey A.M., Chamberlin P., Christman K.L. (2019). First-in-Man Study of a Cardiac Extracellular Matrix Hydrogel in Early and Late Myocardial Infarction Patients. JACC Basic Transl. Sci..

